# Supplementary material for: Ediacaran biozones identified with network analysis provide evidence for pulsed extinctions of early complex life
Source: Nat Commun. 2019 Feb 22;10:911. doi: 10.1038/s41467-019-08837-3 (PMC6384941; doi:10.1038/s41467-019-08837-3)
Supplement: Supplementary file 1 — Supplementary Information [file 41467_2019_8837_MOESM1_ESM.pdf]

Supplementary Information

Ediacaran biozones identified with network analysis provide evidence for pulsed extinctions of early complex life

Muscente et al.

SUPPLEMENTARY DISCUSSION

Supplementary Discussion of Community Detection Algorithms

We applied a number of community detection algorithms to the networks in order to explore their community structures and identify paleocommunities, biotopes, and biozones. Network partitioning algorithms differ with regard to the definition of community (See Supplementary Table 1), and the effectiveness of any one algorithm may fluctuate with certain global (whole-network) properties, like network size (number of nodes) or mixing (i.e. the numbers of links between nodes belonging to different communities). Additionally, whereas some algorithms identify non-overlapping (mutually exclusive) modules, others can detect overlapping community units. As a result, two algorithms may return somewhat different community structures for a given network.

For a number of reasons, we focus on the results returned by the community overlap propagation algorithm (COPRA)[1] (See Supplementary Table 1). First, unlike other algorithms, the method could be applied to both unipartite and bipartite networks[1]. Second, because COPRA exhibits near-linear time complexity for sparse datasets[1], its run times were relatively low. Third, every module identified in a bipartite network with COPRA included one or more nodes from each of its projections[1], meaning that the method leveraged and integrated data. Fourth, the COPRA method allowed for detection of overlapping community units (e.g. paleocommunities, biotopes, and biozones)[1], which are expected to share entities (i.e. taxa). Lastly, the COPRA method performed better than other community detection algorithms. It consistently identified the non-overlapping community structures with the highest Q values (See Supplementary Figure 5 and Supplementary Table 6), affirming that it performed best at identifying nonrandom associations of nodes. It also typically returned the fewest communities, making it one of the most conservative approaches for partitioning the dataset. For this reason, we can interpret the modules as macro-level community units, representing the largest and most significant associations of nodes.

COPRA[1] represents one of many algorithms that involve an iterative, non-deterministic process called label propagation[2] (See Supplementary Table 1). In general, label propagation entails assigning a unique label to each node and then repeatedly replacing the label of each node with the most common label among its neighbors. If two or more labels occur with equal frequency, a node is randomly assigned one of those labels. After a number of iterations, all members of a module will ultimately possess the same label, and the algorithm stops when it achieves a solution with some termination criterion (e.g. every node attains the label used by the maximum number of neighbors). Notably, because label propagation involves randomization, COPRA is a non-deterministic algorithm, and may produce different solutions across multiple runs on a network. To find the best community structure, we ran COPRA 100,000 times on each network, and recorded the solution with the highest modularity. For bipartite networks, we selected the solutions with the highest modularity scores for paleoenvironment and formation projections. We use these projections because environments and formations are larger than organisms and taxa.

The COPRA method innovates on the traditional process of label propagation in two ways. First, the method features a special integrative process for analyzing bipartite networks. Essentially, in bipartite network analysis, node labels are passed from the first projection to the second, and vice versa, back and forth over many iterations. As a result, community units propagate from one projection to the next, until the algorithm identifies the best solution for the

union of data. Second, it incorporates a belonging coefficient in the update step, which allows for nodes to accept multiple labels, and therefore, to belong to several modules. The amount of overlap depends on a user-defined parameter (v), the permissible (maximum) number of communities per node. Since the actual numbers of communities in our networks are unknown, we selected v parameters by jackknife resampling and partitioning networks. Our work assumes that a node could, in theory, be assigned to every module in its network, even if those communities did not otherwise overlap. This assumption accommodates taxa, which could belong to numerous modules if, for example, they existed over long intervals of geologic time, inhabited various environments, and/or were preserved under diverse conditions.

Supplemental Discussion of Modules Identified with COPRA

Although each paleoenvironment was assigned to exactly one biotope, some taxa and formations were assigned to multiple modules. Cases of overlap indicate that nodes could not be assigned to individual modules based on the available data. In bipartite networks of formations and taxa, common taxa (e.g. *Pteridinium*, *Rangea*, and *Vendotaenia*) may be assigned to multiple modules because their fossils are stratigraphically wide-ranging and span multiple biozones. Rare and uncommon taxa, conversely, may be assigned to multiple biozones because they occur in deposits where no meaningful index fossils are preserved (Fig. 6). Along these same lines, formations may be assigned to multiple biozones because they contain ambiguous assemblages of mixed character or, alternatively, they lack index fossils of sufficient biostratigraphic value.

Hierarchical List of Body Fossil Form Categories and Morphogroups

Prokaryotic and Eukaryotic Algae

Form Category/Morphogroup: Horodyskiomorpha

Modular forms consisting of numerous spherical, ellipsoidal, cylindrical, ring-shaped, or (non-nested) cup-shaped units of roughly equal measure, which are generally regularly spaced and do not distort each other. Structures may be connected by strings.

Form category: Chuariomorpha, Moraniomorpha, & Tawuiomorpha

Morphologically simple spherical, discoidal, rectilinear, and simple curvilinear structures lacking ornamentation, possibly containing numerous substructures of equal measure.

Morphogroup: Chuariomorpha

Spherical or discoidal structures, potentially containing numerous sub-structures, but generally unornamented and lacking evidence of association with other similar spheres or discs.

Morphogroup: Moraniomorpha

Irregularly rounded spherical or discoidal structures lacking ornamentation, generally without wrinkles.

Morphogroup: Tawuiomorpha

Rectilinear to simple curvilinear sausage-shaped forms with broad, rounded termini, usually not twisted and lacking transverse elements.

Form category: Eoholyniomorpha, Glomulomorpha, Grypaniomorpha, Longfengshaniomorpha, & Mezeniomorpha

Filamentous, branching, and flat clavate thalli.

Morphogroup: Eoholyniomorpha

Slender (often bush-shaped) thalli that regularly branch. In comparison to Vendotaeniomorphs, these forms are delicate, commonly branch, and show no evidence of twisting.

Morphogroup: Glomulomorpha

Dense aggregates of fasciculate filaments, which may branch; aggregates do not resemble coherent blade-, vesicle, or tube-shaped thalli. In *Longifuniculum*, the aggregate is a fan- or dumbbell-shaped thallus, consisting of a bundle of twisted filaments that flare toward one or both ends.

Morphogroup: Grypaniomorpha

Slender, curvilinear filaments with pronounced coiling tendency and kinked filaments when drawn out.

Morphogroup: Longfengshaniomorpha

Clavate forms with ellipsoidal, ovoidal, or panduroidal (fiddle-shaped vesicles), possibly made up of filaments or fibers, subtended by stalks.

Morphogroup: Mezeniomorpha

Clavate non-branching forms, generally with massive (thick and wide) coherent blades, possibly made up of filaments or fibers, presumably with prominent globose or rhizoidal holdfast (as observed in *Baculiphyca*).

Complex eukaryotes & possible animals

Form Category / Morphogroup: Anhuiphytomorpha

Clavate forms with coherent cone-, fan-, or spindle-shaped thalli, which taper on one or both sides and are sometimes composed of longitudinal fibers or filaments, sometimes with septation. If thalli comprised of filaments, filaments may rarely branch.

Form Category / Morphogroup: Vendotaeniomorpha

Long tube- and ribbon-shaped forms, smooth or patterned, in some cases twisted, possibly comprised of longitudinal fibers or filaments, rarely branching, lacking annulations or segmentation.

Form Category: Sabelliditomorpha, Shaanxilithomorpha, & Sinosabelliditomorpha

Annulated and/or segmented tube-, cylinder-, and ribbon-shaped forms

Morphogroup: Sabelliditomorpha

Long, slender tube- and ribbon-shaped forms with regularly spaced, narrow transverse annulations or segmentation.

Morphogroup: Shaanxilithomorpha
Tubes or cylinders, annulated in appearance, comprised of serially arranged but weakly articulated modules. Disarticulation of these modules results in discoidal, imbricated, meniscate, and lensoidal fossils.

Morphogroup: Sinosabelliditomorpha

Long, slender tube- and ribbon-shaped tomaculate forms with regularly spaced, narrow transverse annulations or segmentation and rounded terminations.

Putative metazoans

Form Category: Anabaritidae, Cloudinomorpha, Namacalathomorpha, Platysolenitomorpha, & Protolagenomorpha

Skeletal forms.

Morphogroup: Anabaritidae

Taxa in the family Anabaritidae, characterized by tubular biomineralized skeletons with trifold symmetry.

Morphogroup: Cloudinomorpha

Tubular and conotubular forms with the appearance of nested (perhaps funnel-shaped) cylinders.

Morphogroup: Namacalathomorpha

Unbranched tubes, each tapering toward one (presumably apical) end and opening up to a cup at the other.

Morphogroup: Platysolenitomorpha

Agglutinated skeletons shaped like tubes.

Morphogroup: Protolagenomorpha

Agglutinated skeletons shaped like vases.

Form Category/Morphogroup: Porifera (putative)

Forms resembling sponges, and encompassing a wide array of morphologies, ranging from discs (e.g. *Coronacollina* and *Palaeophragmodictya*) and encrusters (e.g. *Namapoikia*) to filaments (e.g. *Cucullus)* and cones (e.g. *Thectardis*). In many cases, interpretations of these forms as sponges have been questioned[3,4].

Ediacara-type elements that include possible crown- and stem-group animals

Form Category: Bilateralomorpha, Dickinsoniomorpha, & Kimberellomorpha

Forms with bilateral (or pseudo-bilateral) symmetry and anterior-posterior differentiation.

Morphogroup: Bilateralomorpha

Segmented forms with distinct bilateral symmetries along their lengths, which may be original features or a consequence of their taphonomy. Anterior-posterior differentiation is conspicuous, as these forms have distinct headshield-like anterior regions and repeatedly segmented posterior regions. Segments typically taper in size toward the posterior end. These segments may have been unattached or attached to each other and may have been surround by exterior membranes. Segments may have alternated, rather than opposed each other, across the midline. If so, such forms may not have been bilaterally symmetrical.

Morphogroup: Dickinsoniomorpha

Oval-shaped forms composed of smooth, featureless tubes radiating from a midline that connects the anterior and posterior ends.

Morphogroup: Kimberellomorpha

Oval-shaped, bilaterally symmetrical fossils with several morphologically distinct and concentrically-arranges zones, including an outer smooth and crenulated zone and an inner zone bordered by thin transverse wrinkles and containing a deep longitudinal invagination. The oval shape narrows near one end, thought to represent the anterior end of the organism.

Form Category: Pentaradialomorpha, Tetraradialomorpha, & Triradialomorpha

Forms characterized by three or more lines of symmetry, often in the form of arm-like structures at their centers.

Morphogroup: Pentaradialomorpha

Forms with pentaradial symmetry, such as, circular discs with star-shaped structures of arms at their centers (e.g. *Arkarura*).

Morphogroup: Tetraradialomorpha

Forms with tetraradial symmetry, such as four-lobed radial bodies (e.g. *Conomedusites*).

Morphogroup: Triradialomorpha

Forms with triradial symmetry (e.g. *Anfesta*, *Triforillonia*, and *Tribrachidium*).

Form Category/Morphogroup: Arboreomorpha

Frondose forms with prominent central stalks and bifoliate petaloids. The forms generally consist of parallel primary branches that diverge from the central stalk at acute to right angles (45°–90°) and terminate at an outer margin. The branches are connected, possibly attached to a dorsal sheet. Primary branches consist of teardrop-shaped secondary branches, which are positioned at right angles to the primary branches.

Form Category/Morphogroup: Rangeomorpha & "Ivesheadiomorpha"

Modular, frondose forms composed of leaf-like structures ("frondlets"). The frondlets are repeatedly branched in self-similar patterns that are identical through three or more orders of branching. "Ivesheadiomorpha" is a problematic group of

taxa that often cooccur with rangeomorphs. Given the poor preservation of their fossils, it is possible (if not likely) that ivesheadiomorphs are taphonomically degraded rangeomorphs, in which case, those taxa should be treated as taphomorphs (i.e. junior synonyms or unnamed rangeomorphs).

Form Category/Morphogroup: Erniettomorpha

Diverse modular forms that are smooth, unbranched, and cylindrical in shape. These forms include multi-foliate taxa, each consisting of three or more identical leaf-like petaloids composed of modular tubes arranged around a central axis. The tubes typically alternate along the central midline, indicating that they are not bilaterally symmetrical.

Form Category: Aspidellomorpha & Beltanelliformomorpha

Ornamented discs and discs occurring in groups.

Morphogroup: Aspidellomorpha

Discoidal forms, which are sometimes attached to stalks and often possess ridges as well as concentric and radiating structures, including external tentacle- and rhizoid-like offshoots, but never show evidence spheroidal or discoidal sub-structures.

Morphogroup: Beltanelliformomorpha

Discoidal or spherical forms found in accumulations of densely spaced individuals of equal or varying sizes, frequently distorting each other.

Problematica

Form Category/Morphogroup: Problematica

All other forms, which due to their characters or lack sufficient diagnoses, do not fall within the other morphogroups or form categories.

Crown-group Metazoa

Form Category: Cambrian Metazoa

Phylum: Arthropoda

Phylum: Brachiopoda

Phylum: Chordata

Phylum: Cnidaria

Phylum: Hyolitha

Phylum: Metazoa (*Incertae sedis*)

Small shelly fossils

Phylum: Mollusca

Phylum: Porifera

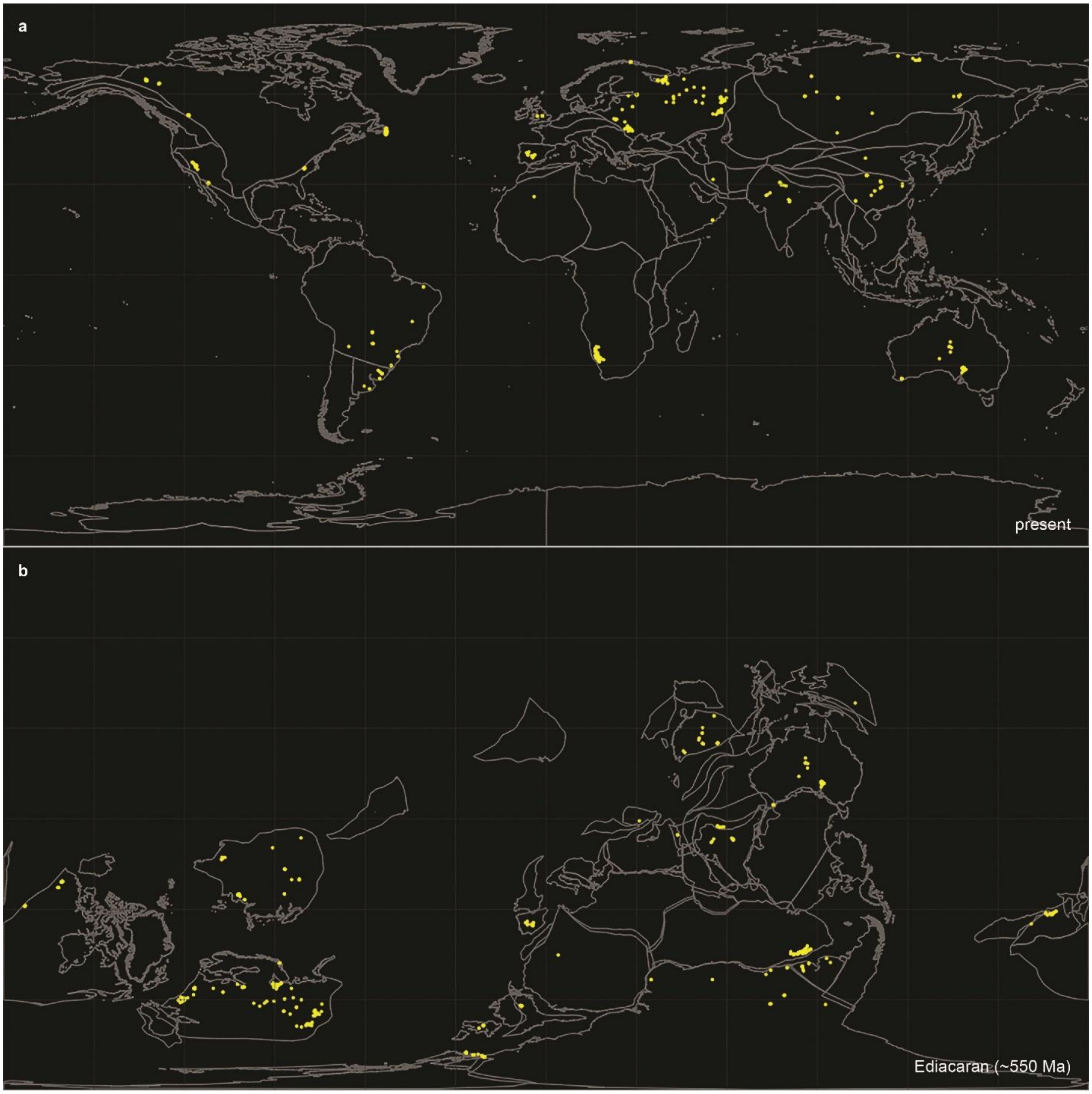

Supplementary Figure 1 | Global maps of Ediacaran fossil collection points included in the original dataset of this study. (**a**) Present locations. (**b**) Locations of sampling points during Ediacaran times around the transition between the Ediacara biota and Terminal Ediacaran biozones. Maps were produced with GPlates. Source data are provided as a Source Data file.

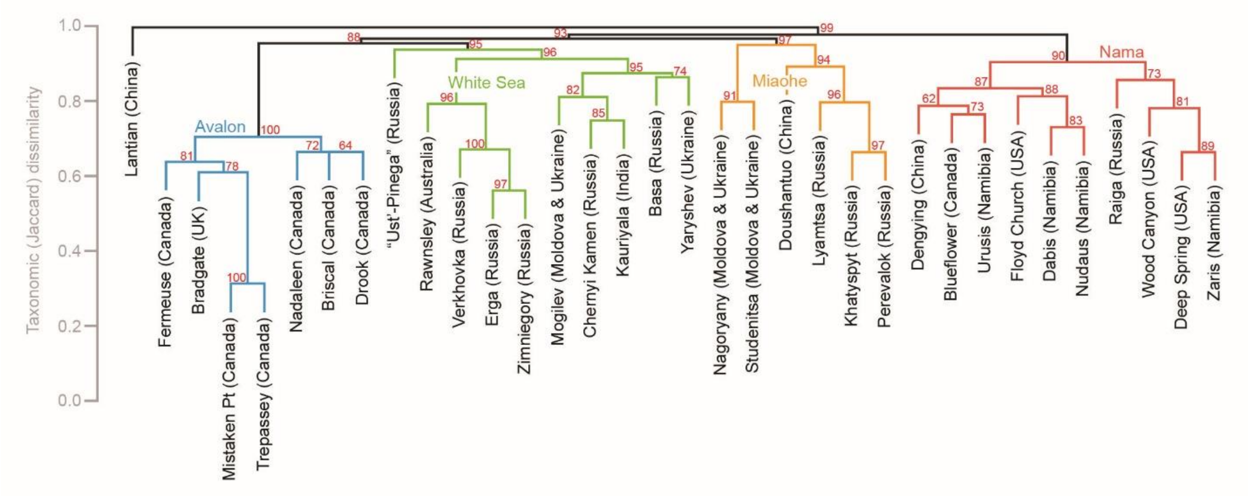

Supplementary Figure 2 | Hierarchical clustering using Jaccard dissimilarities. Dendrogram of 34 geologic formations containing five or more macrofossil genera and/or ichnogenera of Ediacaran age. Formations were clustered based on their taxonomic (Jaccard) dissimilarities[5] (x-axis) using the average-linkage method[6]. The high cophenetic correlation score of the dendrogram (0.8992476)—the correlation between observed taxonomic dissimilarities and those estimated via hierarchical clustering—indicates that the dendrogram faithfully preserves the pairwise distances between the original data points. Red numbers are approximately unbiased (AU) P values calculated for nodes from 1000 multiscale bootstrap resamples. From left to right after the Lantian Formation, the labeled Avalon (blue), White Sea (green), Miaohe (orange), and Nama (red) clusters have AU P values ≥ 0.90 and are supported by the data. The "Ust'-Pinega" formation encompasses occurrences of taxa that belong to the Lyamtsa, Verkhovka, and Zimniegory formations but have uncertain stratigraphic position. Source data are provided as a Source Data file.

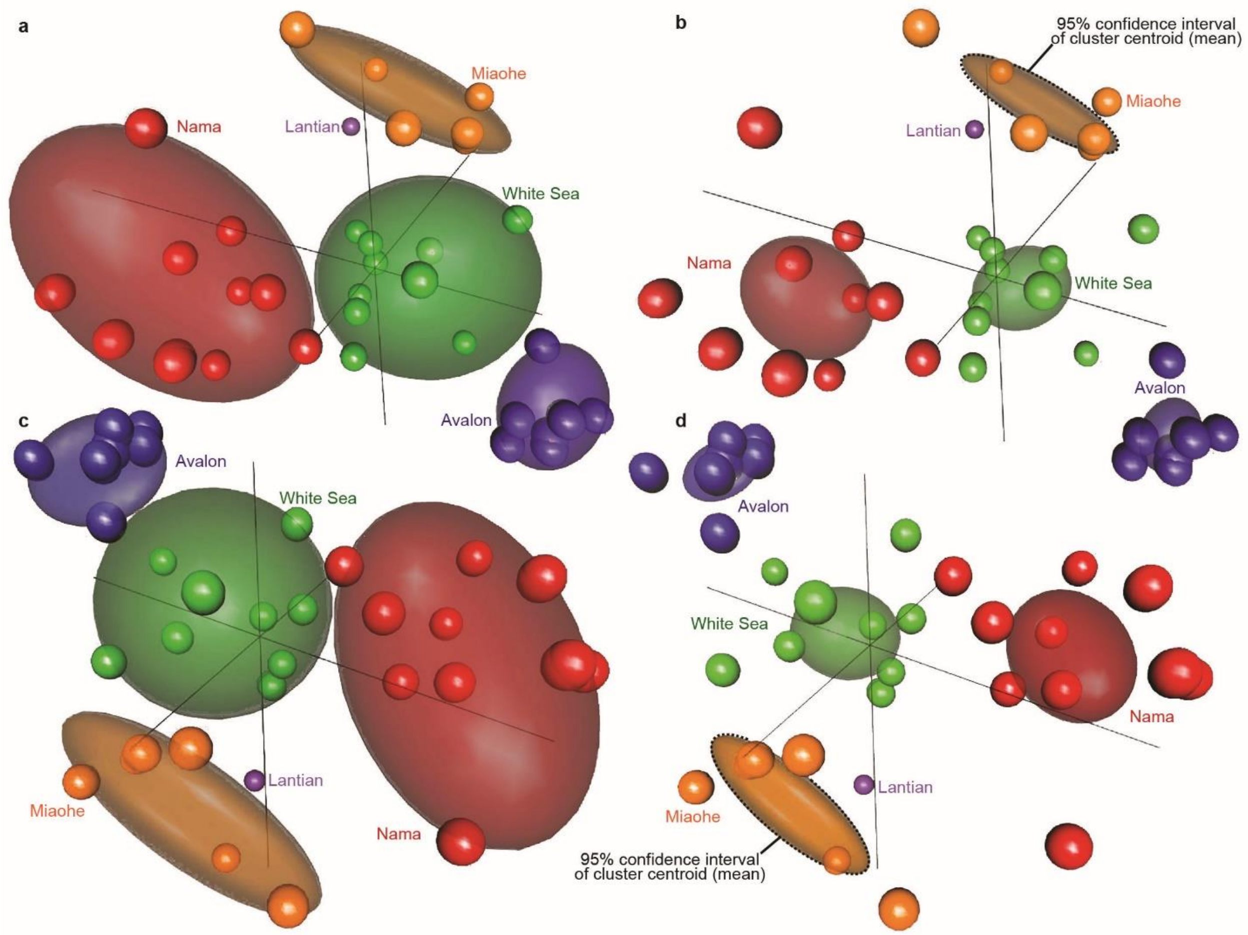

Supplementary Figure 3 | NMDS results. Ordination plots of 34 geologic formations containing five or more macrofossil genera and/or ichnogenera of Ediacaran age. (**a, b**) Kulczynski-2 dissimilarity-based distance results. (**c, d**) Jaccard dissimilarity-based distance results. The k values (numbers of axes) were selected based on the associated stress values (a: 0.121614; b: 0.1190979), which in general, indicate that the plots provide good representations of rank orders in reduced dimensions. In comparison, the k=2 stress values (a: 0.1782327; b: 0.1806682) indicate that two-axis plots would provide relatively poor representations of the data. (**a, c**) Ellipsoid hulls enclose the Avalon, White Sea, Miaohe, and Nama clusters (Fig. 1; See Supplementary Figure 2). (**b, d**) Ellipsoid hulls enclose the 95% confidence intervals around the centroid (means) values of clusters. The centroid ellipses do not overlap, indicating that taxonomic dissimilarities among the clusters are statistically significant. Source data are provided as a Source Data file.

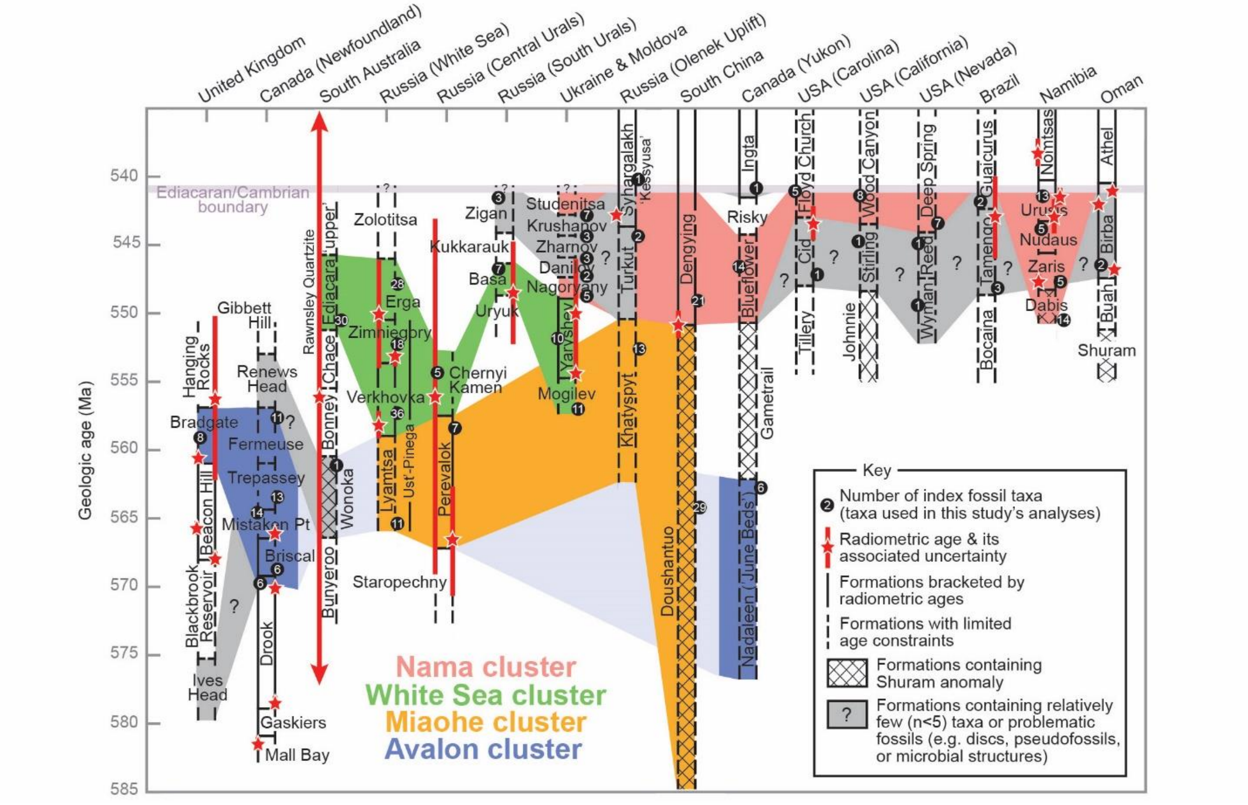

Supplementary Figure 4 | Stratigraphic distribution of Ediacaran macrofossil clusters identified with hierarchical clustering and NMDS (Fig. 1; See Supplementary Figures 2 and 3). White formations contain no potential index fossils. Source data are provided as a Source Data file.

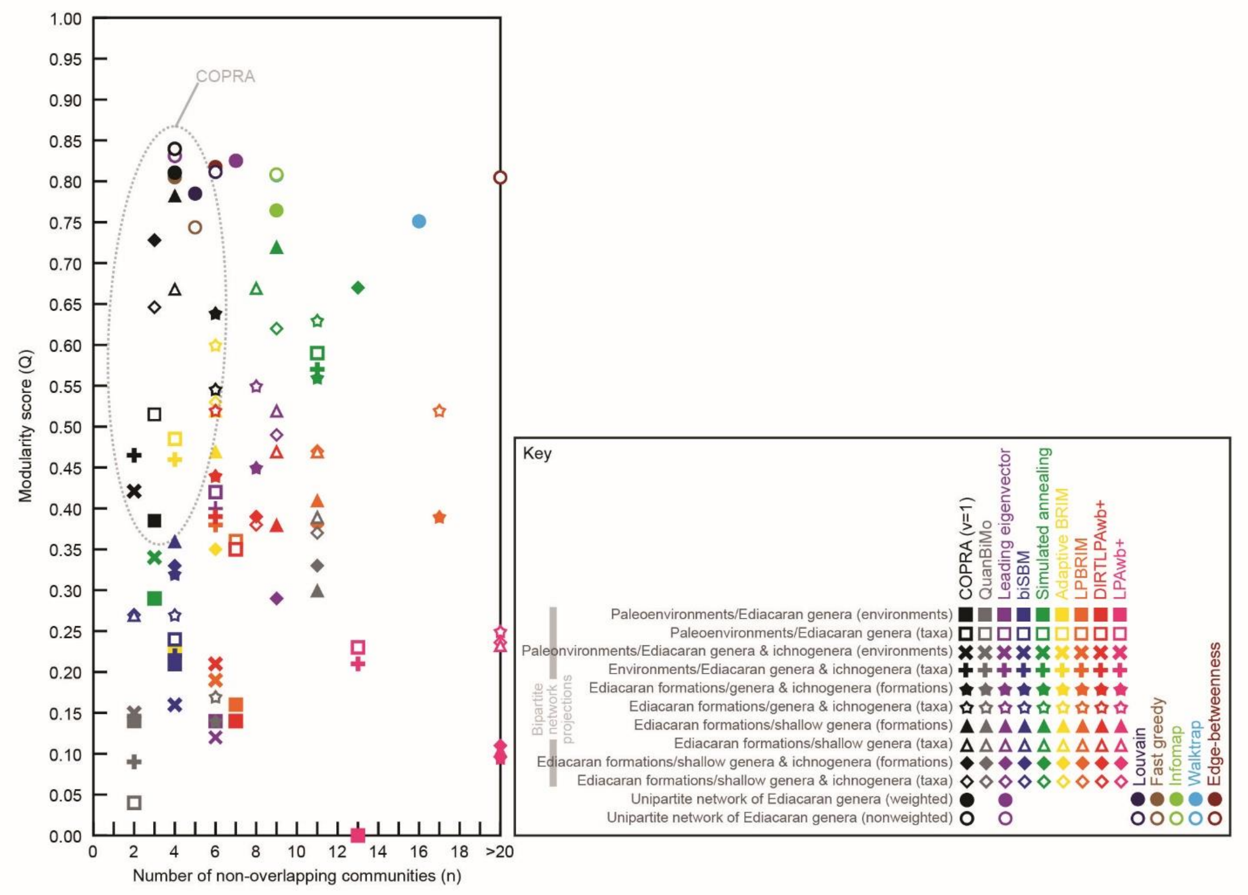

COPRA
Modularity score (Q)
Number of non-overlapping communities (n)
1.00
0.95
0.90
0.85
0.80
0.75
0.70
0.65
0.60
0.55
0.50
0.45
0.40
0.35
0.30
0.25
0.20
0.15
0.10
0.05
0.00
0
2
4
6
8
10
12
14
16
18
>20
Key
COPRA (v=1)
QuanBiMo
Leading eigenvector
biSBM
Simulated annealing
Adaptive BRIM
LPBRIM
DIRTLPAwb+
LPAwb+
Bipartite network projections
Paleoenvironments/Ediacaran genera (environments)
Paleoenvironments/Ediacaran genera (taxa)
Paleoenvironments/Ediacaran genera & ichnogenera (environments)
Environments/Ediacaran genera & ichnogenera (taxa)
Ediacaran formations/genera & ichnogenera (formations)
Ediacaran formations/genera & ichnogenera (taxa)
Ediacaran formations/shallow genera (formations)
Ediacaran formations/shallow genera (taxa)
Ediacaran formations/shallow genera & ichnogenera (formations)
Ediacaran formations/shallow genera & ichnogenera (taxa)
Unipartite network of Ediacaran genera (weighted)
Unipartite network of Ediacaran genera (nonweighted)
Louvain
Fast greedy
Infomap
Walktrap
Edge-betweenness

Supplementary Figure 5 | Results of various network partitioning methods. Plot shows results from fourteen community detection algorithms applied to the networks (See Supplementary Table 6). Weighted and non-weighted versions of the unipartite network were partitioned with the leading eigenvector, Louvain, fast greedy, infomap, walktrap, and edge-betweenness algorithms in the igraph package of R[7], in addition to the COPRA method (v=1) of the COPRA software[1]. Non-weighted versions of the bipartite networks were partitioned with the COPRA method (v=1) [1]; QuanBiMo, LPAwb, and DIRTLPAwb algorithms of the bipartite package in R[8]; LP-BRIM algorithm of the lpbrim package produced by T. Poisot and D. B. Stouffer (http://poisotlab.io/software/) for R; simulated annealing algorithm of the rnetcarto package produced by G. Doulcier, R. Guimera, and D. B. Stouffer for R; leading eigenvector and Adaptive BRIM algorithms of the BiMat package in MATLAB[9]; and biSBM algorithm of the C++ code made available by D. Larremore (http://danlarremore.com/bipartiteSBM/)[10]. Each algorithm outputs a community structure by dividing the nodes of a given network among modules of number (n). The algorithms, in essence, differ with regard to the definition of community, and sometimes partition networks into different numbers of modules. Each algorithm outputs a single best fit community structure, except for the COPRA, LPAwb, DIRTLPAwb, LPBRIM, Adaptive BRIM, biSBM, and QuanBiMo methods, which involve randomization processes and may produce any number of outputs for a given network. With the exception of biSBM, which produced results that did not greatly vary run to run, these methods lacking output determinism were repeatedly applied to each network, and the outputs with the best modularity scores were saved. The QuanBiMo algorithm was run 100 times; the Adaptive BRIM algorithm was run 1000 times; the LPBRIM algorithm was run 10,000 times; and the COPRA, LPAwb, and DIRTLPAwb algorithms were run 100,000 times. The x-axis is the number of non-overlapping modules, and the y-axis is the extended modularity score (Q)[11] of the community structure of the best output. Modularity is a measure of the strength of the division of a community structure. For a given set of communities, Q is the fraction of links that connect nodes of the same communities minus the corresponding fraction expected in an equivalent network with a random distribution of connections. An ellipse has been drawn around the values produced by the COPRA algorithm to illustrate its results in comparison to those of other algorithms. Source data are provided as a Source Data file.

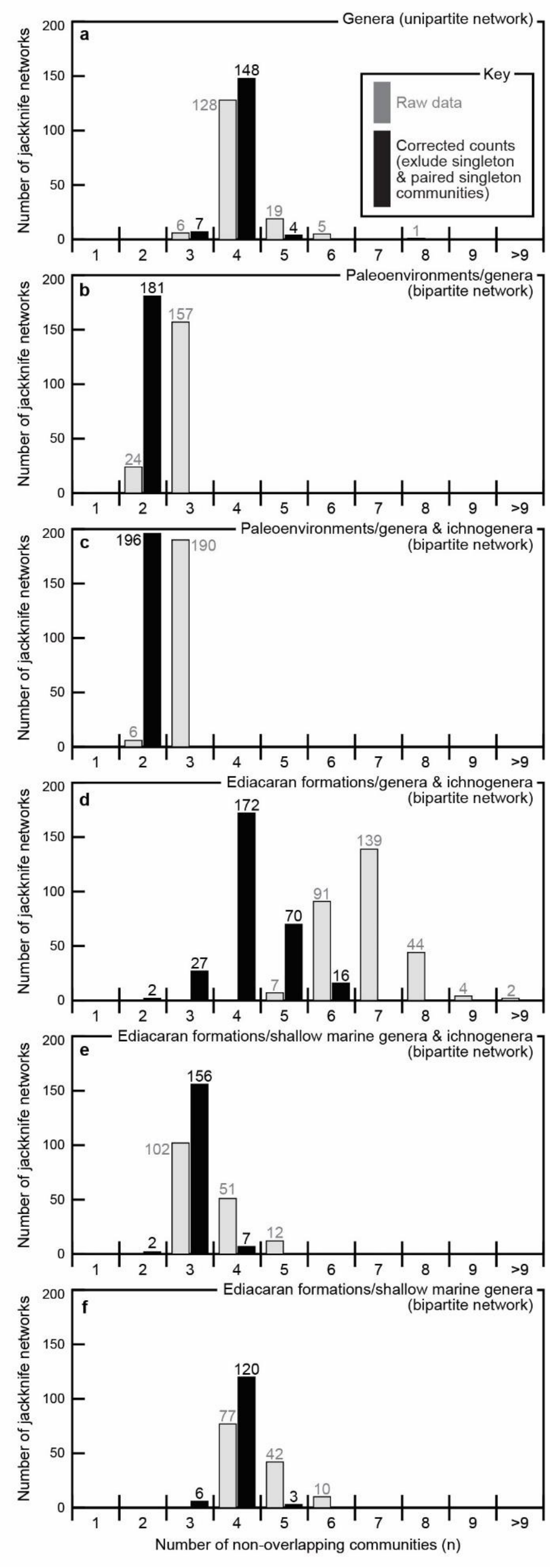

Supplementary Figure 6 | Jackknife network partitioning results. The COPRA method allows for detection of overlapping community structure, in which, nodes may belong to multiple modules. The amount of overlap depends on a user-defined parameter (v), which equals the maximum number of communities per node. To determine *v* parameters, the following jackknife resampling procedure was applied to each network. A single node was removed, the network was partitioned using the COPRA method (*v*=1), and the number of non-overlapping communities (*n*) was recorded. The node was then reinserted into the network, and the steps were systematically repeated, so every node in the network was omitted once and a distribution of *n* values was produced. The number of *n* values in this distribution equals the number of nodes in the network. The distribution is presented as the *n* value (x-axis) versus the number of jackknife networks produced via resampling (y-axis). The raw numbers of jackknife networks are shown along with corrected counts, which exclude singleton and pair singleton communities. In unipartite networks (**a**), singleton communities are solitary nodes, and in bipartite networks (**b**–**f**), pair singleton communities are modules where one or both projections are represented by singletons. (**a**) Unipartite network of Ediacaran macrofossil genera (Fig. 2). (**b**) Bipartite network of paleoenvironments and Ediacaran genera (Fig. 3). (**c**) Bipartite network of paleoenvironments and Ediacaran genera and ichnogenera (See Supplementary Figure 10). (**d**) Bipartite network of Ediacaran formations and genera and ichnogenera (Fig. 4). (**e**) Bipartite network of Ediacaran formations and shallow genera/ichnogenera (See Supplementary Figure 11). (**f**) Bipartite network of Ediacaran formations and shallow genera (See Supplementary Figure 12). For a given network, the v parameter equals the maximum value of *n* in the distribution. Source data are provided as a Source Data file.

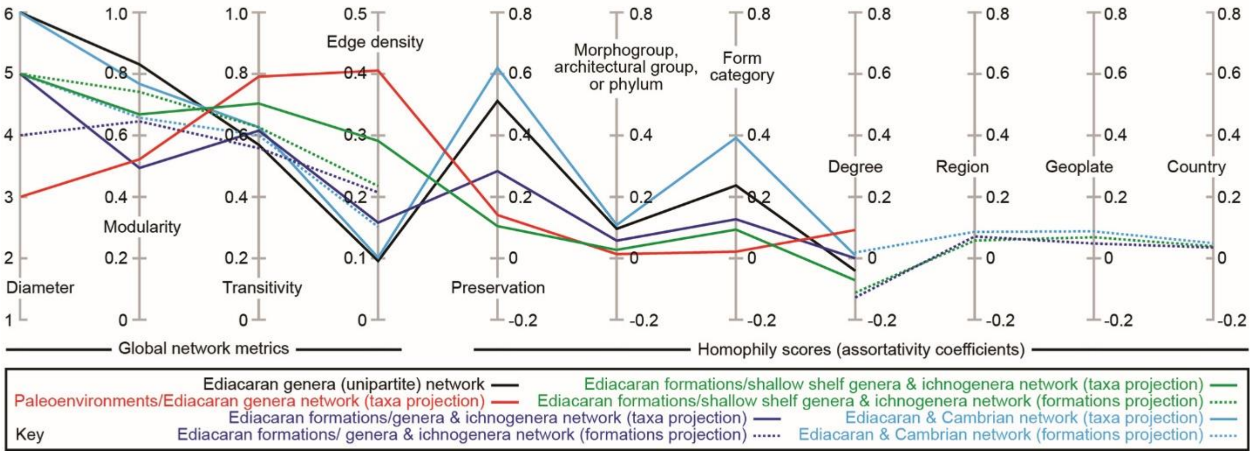

Supplementary Figure 7 | Parallel coordinates plot of network metrics. From left to right, plot shows the values of diameter (maximum degree of separation); modularity (strength of division of network into a particular set of communities); transitivity (triadic closure, i.e. the probability that neighbors of a node are connected); edge density (the ratio between the numbers of actual connections and possible links within a given network); and homophily (the tendency of nodes to associate with others possessing similar properties). The homophily metric (assortativity coefficient) is provided for various nominal variables, including preservational mode (See Supplementary Table 2), morphogroup or phylum (See Supplementary Discussion), form category (See Supplementary Discussion), degree (number of links to other taxa), region (continent), geoplate (tectonic plate), and country. Values (See Supplementary Tables 7 and 8) are provided for the unipartite Ediacaran genera network (Fig. 2), the bipartite Ediacaran formations/genera and ichnogenera network (Fig. 4), the bipartite Ediacaran formations/shallow marine genera and ichnogenera network (See Supplementary Figure 11), the bipartite Ediacaran and Cambrian (formations/genera and ichnogenera) network (See Supplementary Figure 13), and the taxa projection of the bipartite paleoenvironment/Ediacaran genera network (Fig. 3). Source data are provided as a Source Data file.

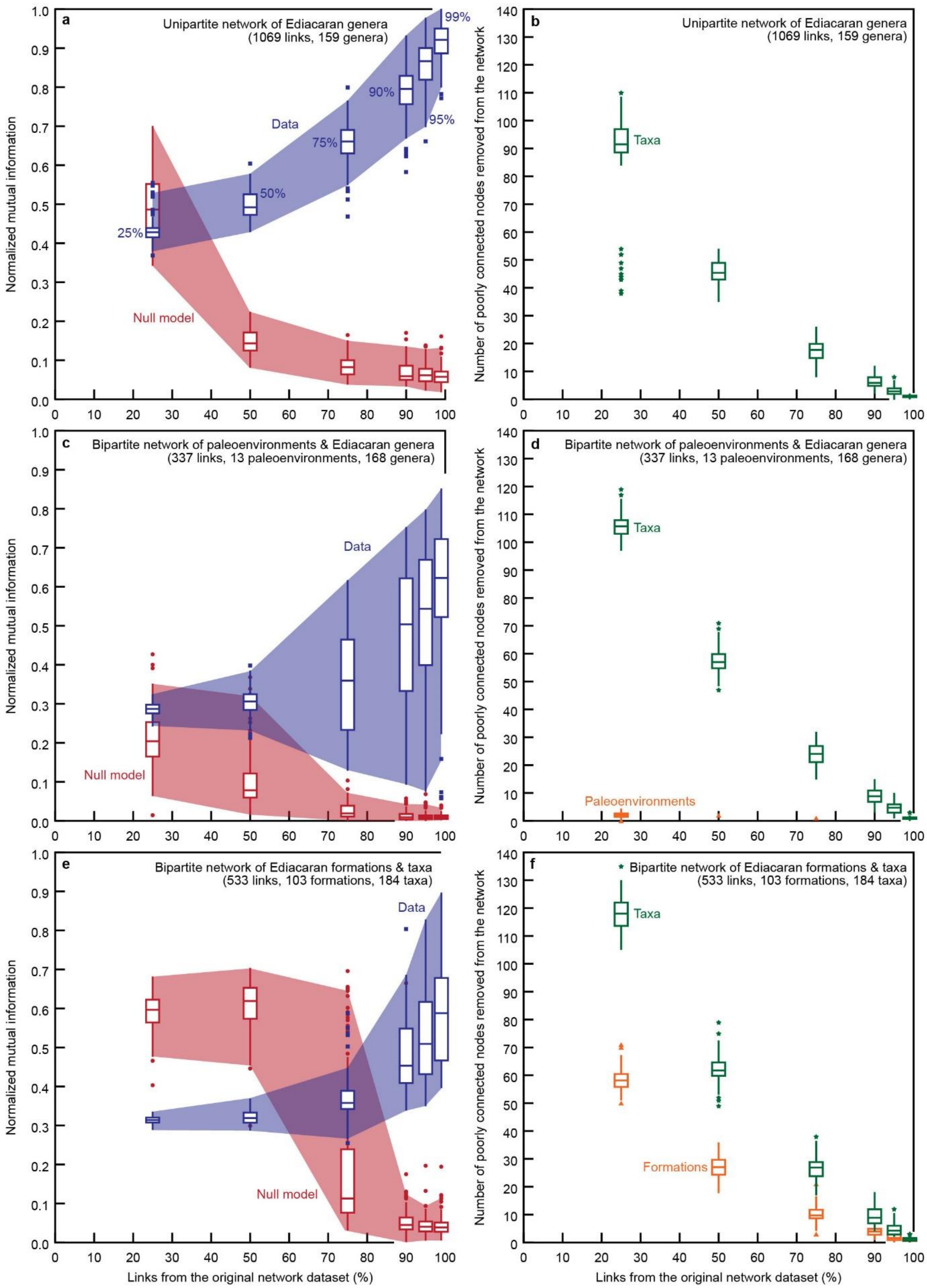
a
Unipartite network of Ediacaran genera
(1069 links, 159 genera)
Data
Null model
25%
50%
75%
90%
95%
99%
Normalized mutual information
b
Unipartite network of Ediacaran genera
(1069 links, 159 genera)
Taxa
Number of poorly connected nodes removed from the network
c
Bipartite network of paleoenvironments & Ediacaran genera
(337 links, 13 paleoenvironments, 168 genera)
Data
Null model
Normalized mutual information
d
Bipartite network of paleoenvironments & Ediacaran genera
(337 links, 13 paleoenvironments, 168 genera)
Taxa
Paleoenvironments
Number of poorly connected nodes removed from the network
e
Bipartite network of Ediacaran formations & taxa
(533 links, 103 formations, 184 taxa)
Data
Null model
Normalized mutual information
Links from the original network dataset (%)
f
Bipartite network of Ediacaran formations & taxa
(533 links, 103 formations, 184 taxa)
Taxa
Formations
Number of poorly connected nodes removed from the network
Links from the original network dataset (%)

Supplementary Figure 8 | Sensitivity analysis. An analysis was conducted to assess the sensitivity of the network partitioning results to the level of sampling of links and nodes. (**a, b**) Unipartite network of Ediacaran genera (Fig. 2a). (**c, d**) Bipartite network of paleoenvironments and Ediacaran genera (Fig. 3a). (**e, f**) Bipartite network of Ediacaran formations and taxa (Fig. 4a). (**a, c, e**) Box-and-whisker plots of normalized mutual information (NMI) scores versus sampling level. Sampling level is the percentage of links that were randomly sampled from the network in order to specify a subnetwork. Each box-and-whisker plot illustrates a distribution of one hundred NMI scores. Each score compares the community structure of the network to the community structure of a subnetwork. Where NMI scores are high, networks and subnetworks have similar community structures and results are relatively robust and insensitive to variation in the data. The shaded areas illustrate the results of hypothesis testing with a null model. In this work, the null hypothesis is that a network and its subnetworks do not have similar community structures. Where the majority (95%) of scores calculated from the data are greater than those of the null model, the null hypothesis can be rejected. (**b, d, f**) Box-and-whisker plots of omitted node counts versus sampling intensity. Each box-and-whisker plot represents a distribution of 100 values, and each value is the number of nodes omitted from a subnetwork as a consequence of the subsampling procedure used to generate the NMI scores in (**a, c, e**). For each box-and-whisker plot, the box encompasses the first and third quartiles, the middle bar depicts the median, and the whiskers depict the true minimum and maximum values except where outliers (dots) greater than 1.5 times the interquartile range are present. Source data are provided as a Source Data file.

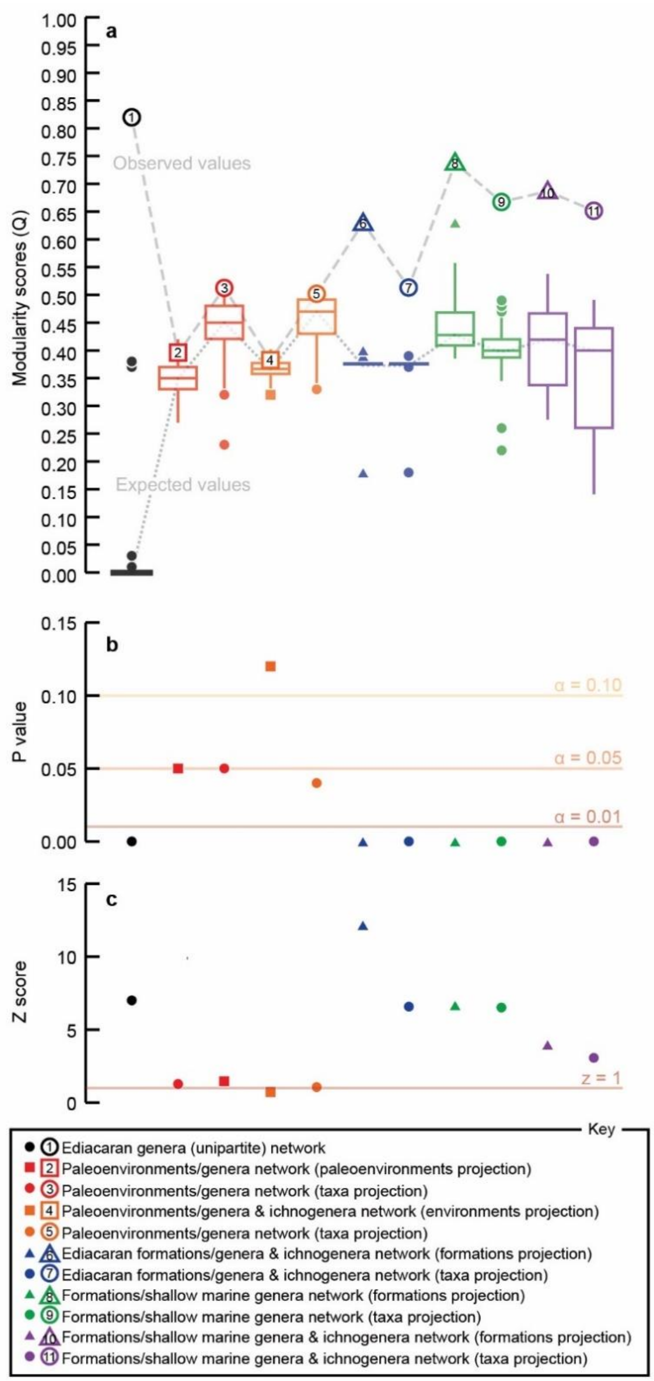

Supplementary Figure 9 | Randomization testing. To determine if the community structures identified with the COPRA community detection algorithm are statistically significant, data randomization tests were conducted. In this work, for unipartite networks, the null hypothesis is that the observed modularity score (*Q*) of a network (See Supplementary Table 7) equals the score of a random network of matching size and degree distribution (the degree of a node equals the number of unique links incident upon it and excludes self-loops and multiple edges). For bipartite networks, the null hypothesis is that the *Q* values of one or both of its projections (See Supplementary Table 7) equal the corresponding score(s) of a random network of matching size and degree distribution. For each network, the links among nodes were randomized, but the nodes' degree distribution was preserved. The randomized network was then partitioned using the COPRA method that was applied to the original network, and the *Q* value of the community structure was recorded. These steps were repeated 100 times for each network, producing one distribution of modularity scores per unipartite network and two distributions of modularity scores (one for each projection) per bipartite network. The distribution of *Q* for each network projection in this study is presented as a box and whisker plot (**a**). The box encompasses the first and third quartiles, the bar in the box depicts the median, and the whiskers show the true minimum and maximum values, except where outliers greater or less than 1.5 times the interquartile range were identified. The distributions were used to calculate P values (**b**) and Z scores (**c**). The P value indicates the probability that a random network projection would have a greater *Q* value. Similarly, its Z score indicates how many standard deviations the observed *Q* is above a random network. If the P value of a unipartite network or both P values of a bipartite network are less than alpha (α) at the 90%, 95%, and/or 99% confidence levels, the null hypothesis can be rejected, and its community structure is considered statistically significant. A Z score greater than 1 also indicates that an observed community structure is significant[12]. Source data are provided as a Source Data file.

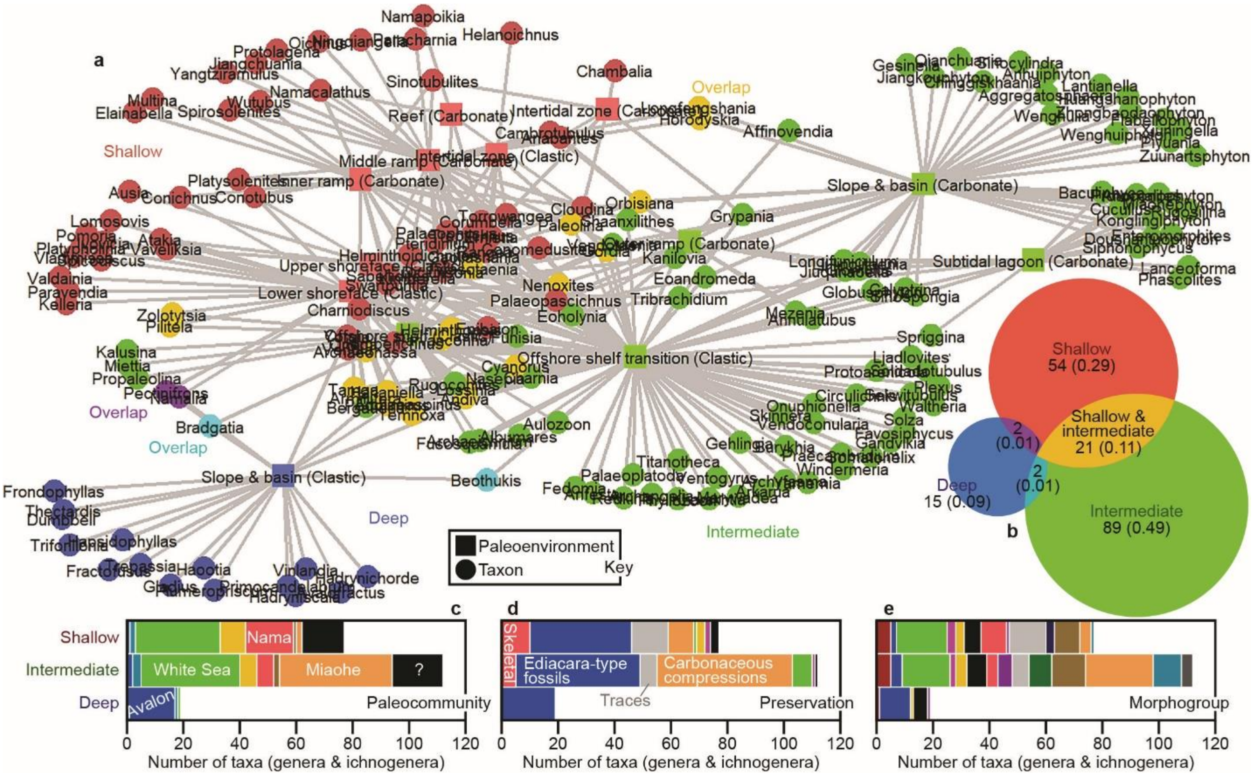

Supplementary Figure 10 | Bipartite network of paleoenvironments and Ediacaran macrofossil taxa. (**a**) Network graph. A taxon (genus or ichnogenus) and paleoenvironment are linked if fossils of the taxon have been reported from matching facies of Ediacaran age. Colors indicate modules identified using the COPRA community-detection algorithm (v=2). According to randomization testing, the community structure is not statistically significant (See Supplementary Figure 9; paleoenvironments projection, Q=0.38, P=0.12, Z=0.73; taxa projection, Q=0.50, P=0.04, Z=1.07) because ichnogenera tie together all modules. All paleoenvironments and taxa fall into three modules—the deep (blue), shallow (red), and intermediate (green) clusters—named for the relative water depths of their centroids along a model shallow-to-deep water transect. (**b**) Venn diagram illustrating taxonomic overlap of modules. Areas of circles correspond to their relative numbers of taxa, numbers are counts of taxa, and values in parentheses are proportions. (**c**) Stacked bar graph showing numbers of genera belonging to the various Ediacaran paleocommunities in each module (colors are those used in Fig. 2a, b). (**d, e**) Stacked bar graphs showing numbers of genera in the modules and their preservational modes (**d**) and morphogroups (**e**). See Fig. 2 for color keys and main text (Fig. 3) for a comparable network that excludes ichnogenera but has a statistically significant community structure. Source data are provided as a Source Data file.

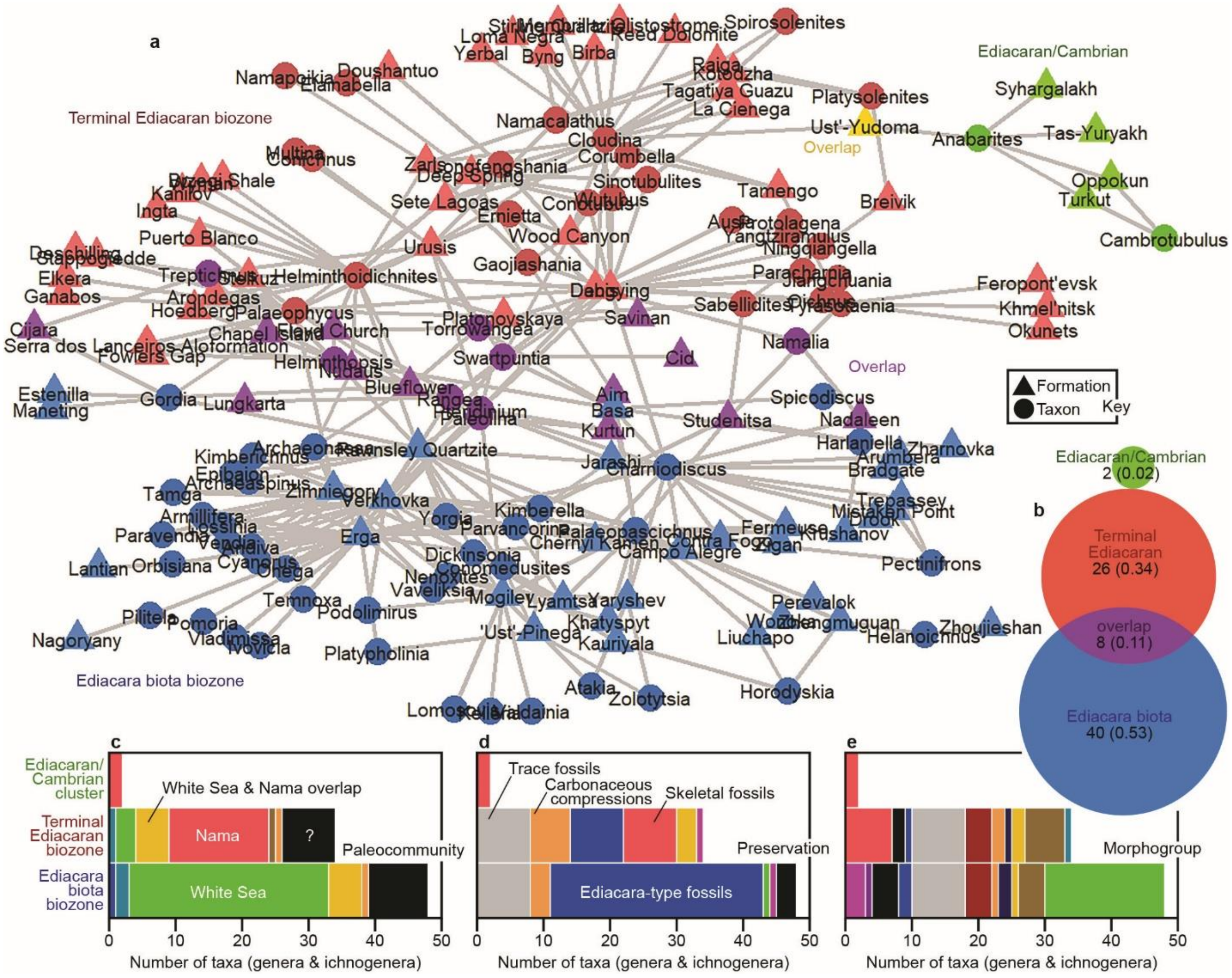

Supplementary Figure 11 | Bipartite network of shallow water macrofossil genera and ichnogenera in Ediacaran formations. (**a**) Network graph. A taxon (genus or ichnogenus) and formation are linked if fossils of the taxon have been reported from that geologic unit. Colors indicate modules identified using the COPRA community-detection algorithm (v=4). According to randomization testing, this community structure is statistically significant (See Supplementary Figure 9; formations projection, Q=0.69, P<0.01, Z=3.98; taxa projection, Q=0.65, P<0.01, Z=3.06). All formations and taxa fall into three modules: the Ediacara biota biozone (blue), the Terminal Ediacaran biozone (red), and the Ediacaran/Cambrian taxa (green) clusters. (**b**) Venn diagram illustrating taxonomic overlap of modules. Areas of circles correspond to their relative numbers of genera and ichnogenera, numbers are counts of taxa, and values in parentheses are proportions. (**c**) Stacked bar graph showing the numbers of taxa belonging to the various Ediacaran macrofossil paleocommunities (colors are those used in Fig. 2a, b) and representing traces in the modules. (**d, e**) Stacked bar graphs showing numbers of taxa in the modules and their preservational modes (**d**) and morphogroups (**e**). See Fig. 2 for color keys. Source data are provided as a Source Data file.

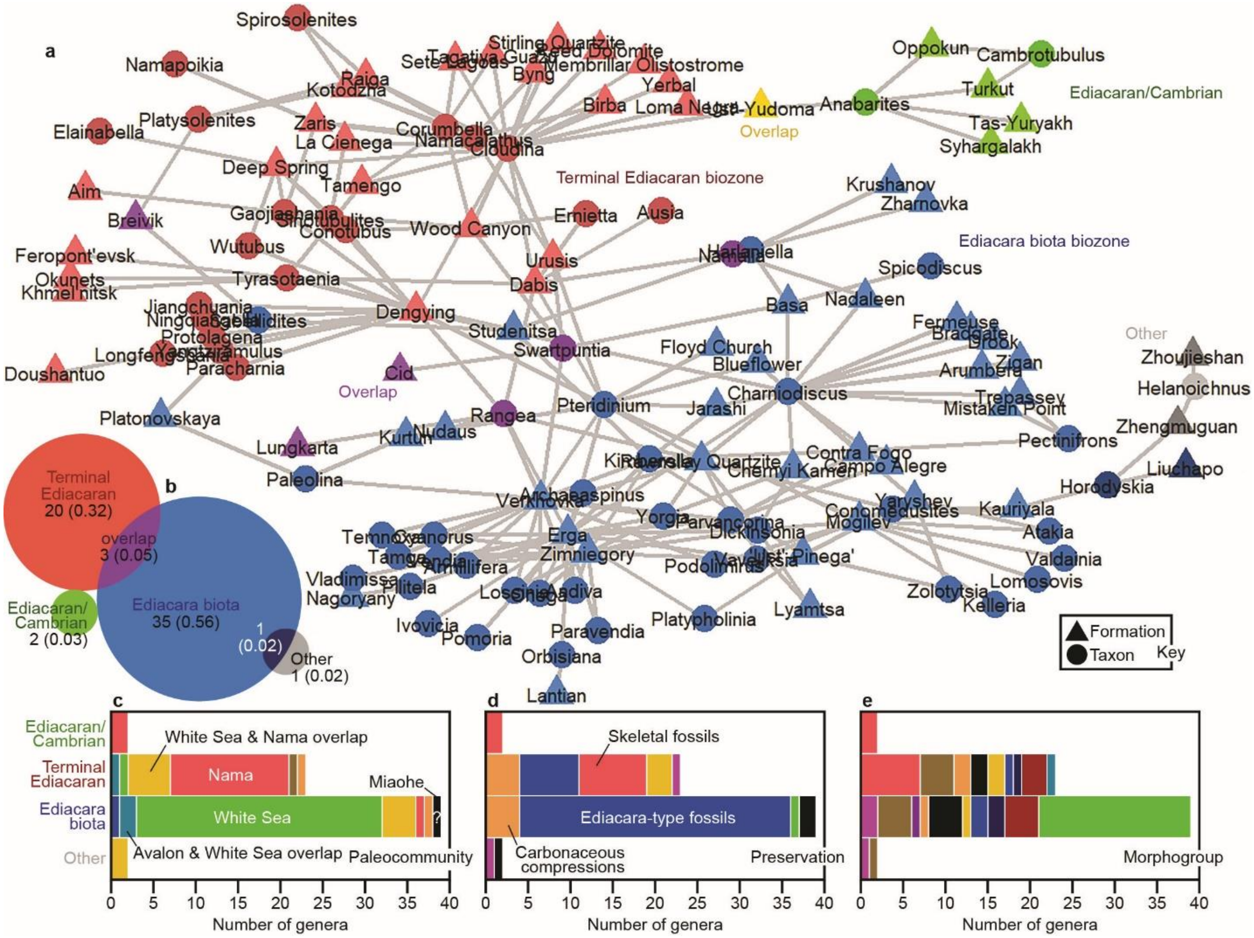

Supplementary Figure 12 | Bipartite network of shallow water macrofossil genera in Ediacaran formations. (**a**) Network graph. A genus and formation are linked if fossils of the taxon have been reported from that geologic unit. Colors indicate modules identified using the COPRA community-detection algorithm (*v*=5). According to randomization testing, this community structure is statistically significant (See Supplementary Figure 9; formations projection, Q=0.74, P<0.01, Z=6.67; taxa projection, Q=0.66, P<0.01, Z=6.51). The majority of formations and genera (98%) fall into three modules: the Ediacara biota biozone (blue), the Terminal Ediacaran biozone (red), and the Ediacaran/Cambrian taxa (green) clusters. (**b**) Venn diagram illustrating taxonomic overlap of modules. Areas of circles correspond to their relative numbers of genera, numbers are counts of taxa, and values in parentheses are proportions. (**c**) Stacked bar graph showing the numbers of genera belonging to the various Ediacaran macrofossil paleocommunities in the modules (colors are those used in Fig. 2a, b). (**d, e**) Stacked bar graphs showing numbers of taxa in the modules and their preservational modes (**d**) and morphogroups (**e**). See Fig. 2 for color keys. Source data are provided as a Source Data file.

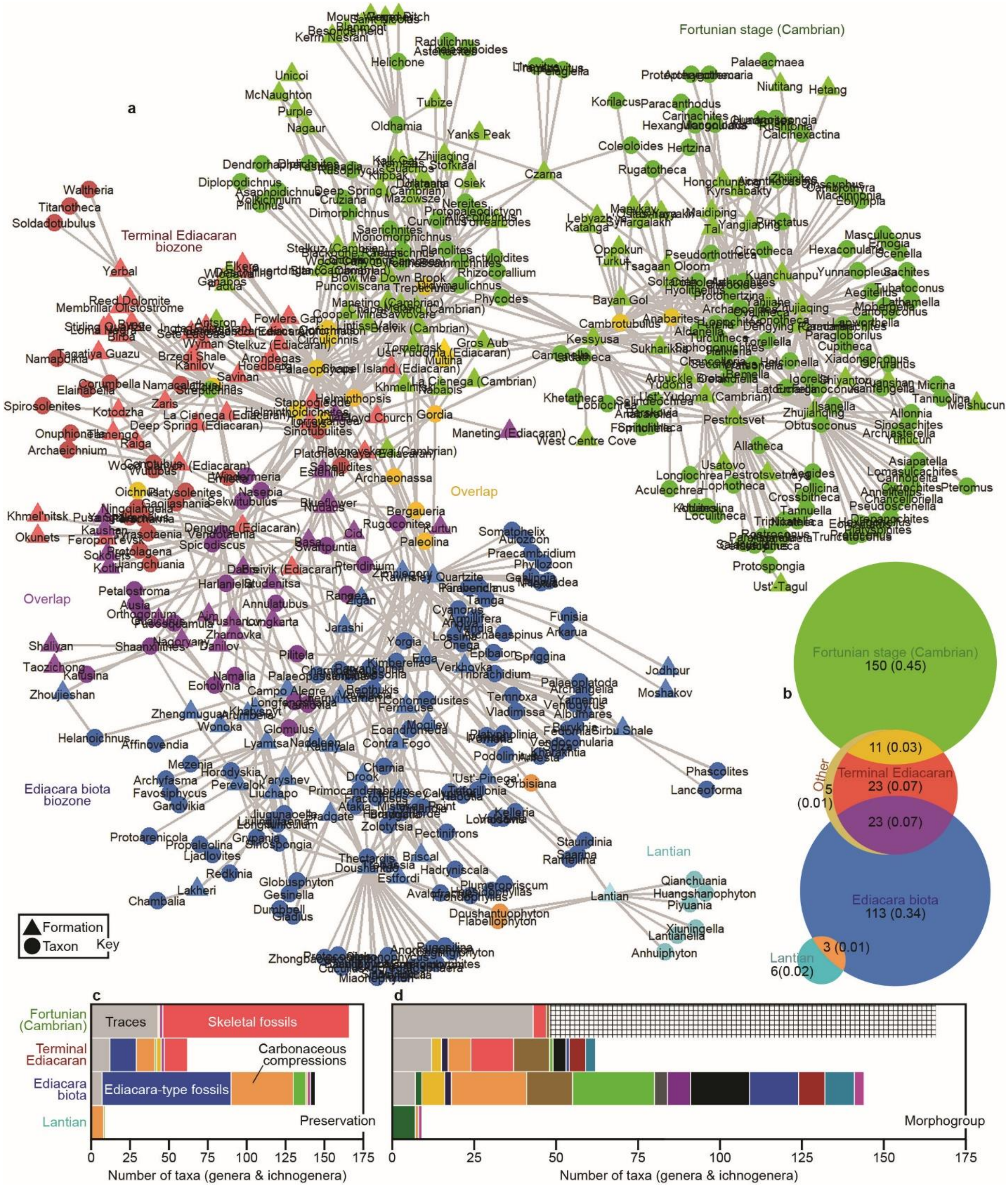
a
Fortunian stage (Cambrian)
Terminal Ediacaran biozone
Overlap
Overlap
Ediacara biota biozone
Lantian
Formation
Taxon
Key
b
Fortunian stage (Cambrian)
150 (0.45)
11 (0.03)
Terminal Ediacaran
23 (0.07)
23 (0.07)
Other
5
(0.01)
Ediacara biota
113 (0.34)
3 (0.01)
Lantian
6(0.02)
c
Fortunian (Cambrian)
Terminal Ediacaran
Ediacara biota
Lantian
Traces
Skeletal fossils
Carbonaceous compressions
Ediacara-type fossils
Preservation
0
25
50
75
100
125
150
175
Number of taxa (genera & ichnogenera)
d
Morphogroup
0
25
50
75
100
125
150
175
Number of taxa (genera & ichnogenera)

Supplementary Figure 13 | Bipartite network of macrofossil taxa and geolologic formations in the Ediacaran System and Fortunian (basal Cambrian) Stage. (**a**) Network graph. The network is identical to the bipartite network of Ediacaran formations and taxa (Fig. 4), except it has been expanded and revised through incorporation of data (see methods) on occurrences of fossils in the Fortunian stage (~541-529 Ma). (**b**) Venn diagram illustrating taxonomic overlap of modules (Ediacaran biozone and Fortunian stage). Areas of circles correspond to their relative numbers of genera and ichnogenera, numbers are counts of taxa, and values in parentheses are proportions. (**c, d**) Stacked bar graphs showing numbers of genera in the modules and their preservational modes (**c**) and morphogroups (**d**). See Fig. 2 for color keys. Source data are provided as a Source Data file.

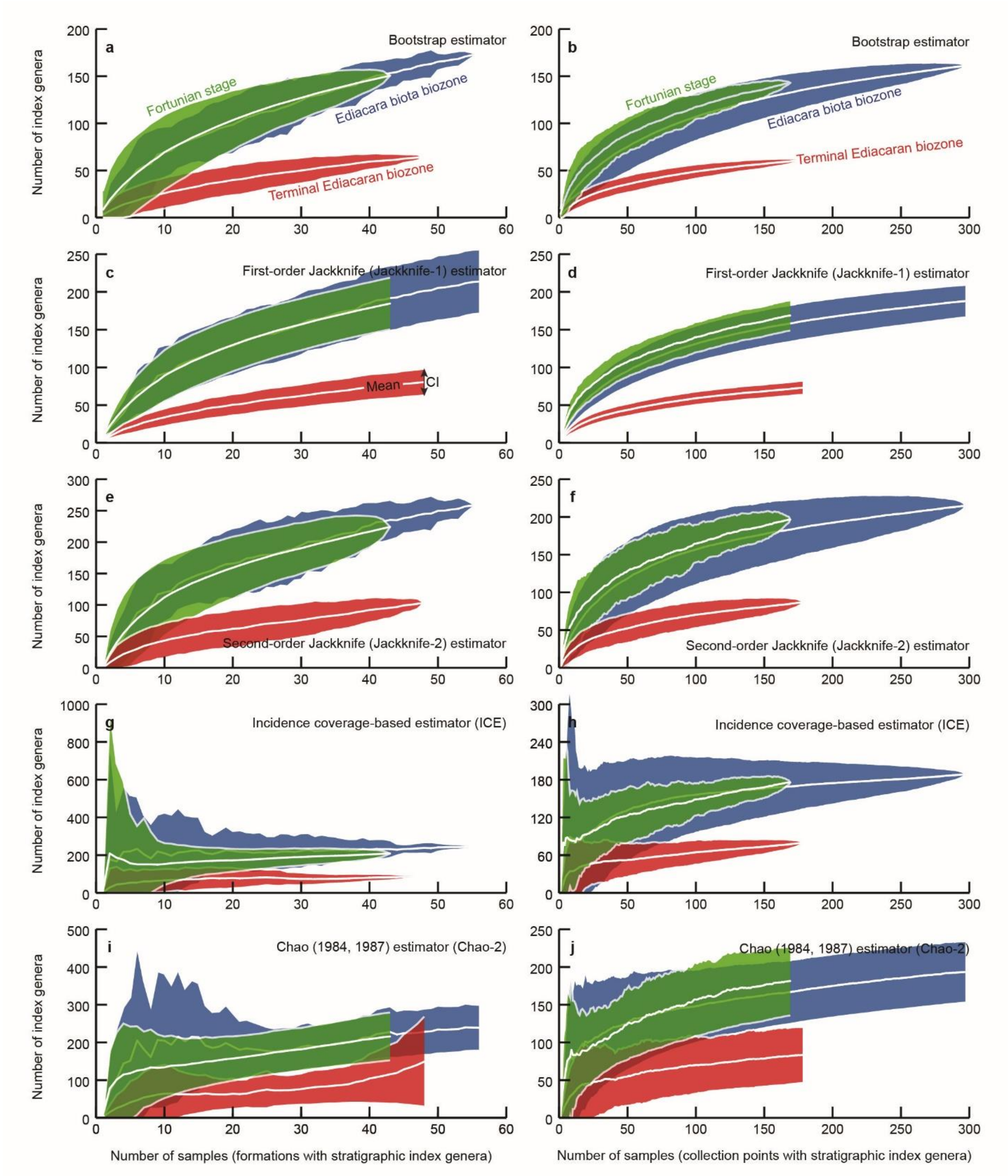
a
Bootstrap estimator
Fortunian stage
Ediacara biota biozone
Terminal Ediacaran biozone
b
Bootstrap estimator
c
First-order Jackknife (Jackknife-1) estimator
Mean
CI
d
First-order Jackknife (Jackknife-1) estimator
e
Second-order Jackknife (Jackknife-2) estimator
f
Second-order Jackknife (Jackknife-2) estimator
g
Incidence coverage-based estimator (ICE)
h
Incidence coverage-based estimator (ICE)
i
Chao (1984, 1987) estimator (Chao-2)
j
Chao (1984, 1987) estimator (Chao-2)
Number of index genera
Number of samples (formations with stratigraphic index genera)
Number of samples (collection points with stratigraphic index genera)

Supplementary Figure 14 | Generic diversity of biozones estimated using non-parametric richness estimators (stratigraphic index fossil genera). Data includes only stratigraphic index fossils (Fig. 4a). Plots show generic diversity (number of genera) versus sampling intensity for the stage-level biozones identified in this study. For a given sampling intensity level, plotted values of diversity are means of 1,000 randomizations of sample order and are bracketed by 95% confidence interval (CI) envelopes. The values were calculated using five different richness estimators[13]. (**a, b**) Bootstrap estimator. (**c, d**) First-order Jackknife (Jackknife-1) estimator. (**e, f**) Second-order Jackknife (Jackknife-2) estimator. (**g, h**) Incidence coverage-based estimator (ICE). (**i, j**) Chao (1984, 1987) estimator (Chao-2). The estimates were calculated from samples defined as geologic formations (**a, c, e, g, i**) and collection points (**b, d, f, h, j**) with relevant body fossils. Collection points were assigned to biozones based on the community assignments of their formations (Fig. 4; See Supplementary Figure 13). Whereas the CIs in (**a, b, e–h**) were calculated from conditional standard deviations determined via stochastic resampling, the CIs in (**c, d, i, j**) were calculated from unconditional standard deviations, which were derived using exact analytical methods[13]. For this reason, the unconditional confidence intervals in (**c, d, i, j**) do not converge to zero variance at the reference sample, like the conditional confidence intervals in (**a, b, e–h**). In general, non-overlap of 95% CIs constructed from unconditional variance estimators (Jackknife-1 and Chao-2) can be used as a simple but conservative criterion of statistical difference[13]. On the other hand, non-overlap of 95% CIs constructed from conditional variance estimators (Bootstrap, Jackknife-2 and ICE) can be used as a criterion that a smaller reference sample was not drawn from a larger one. Source data are provided as a Source Data file.

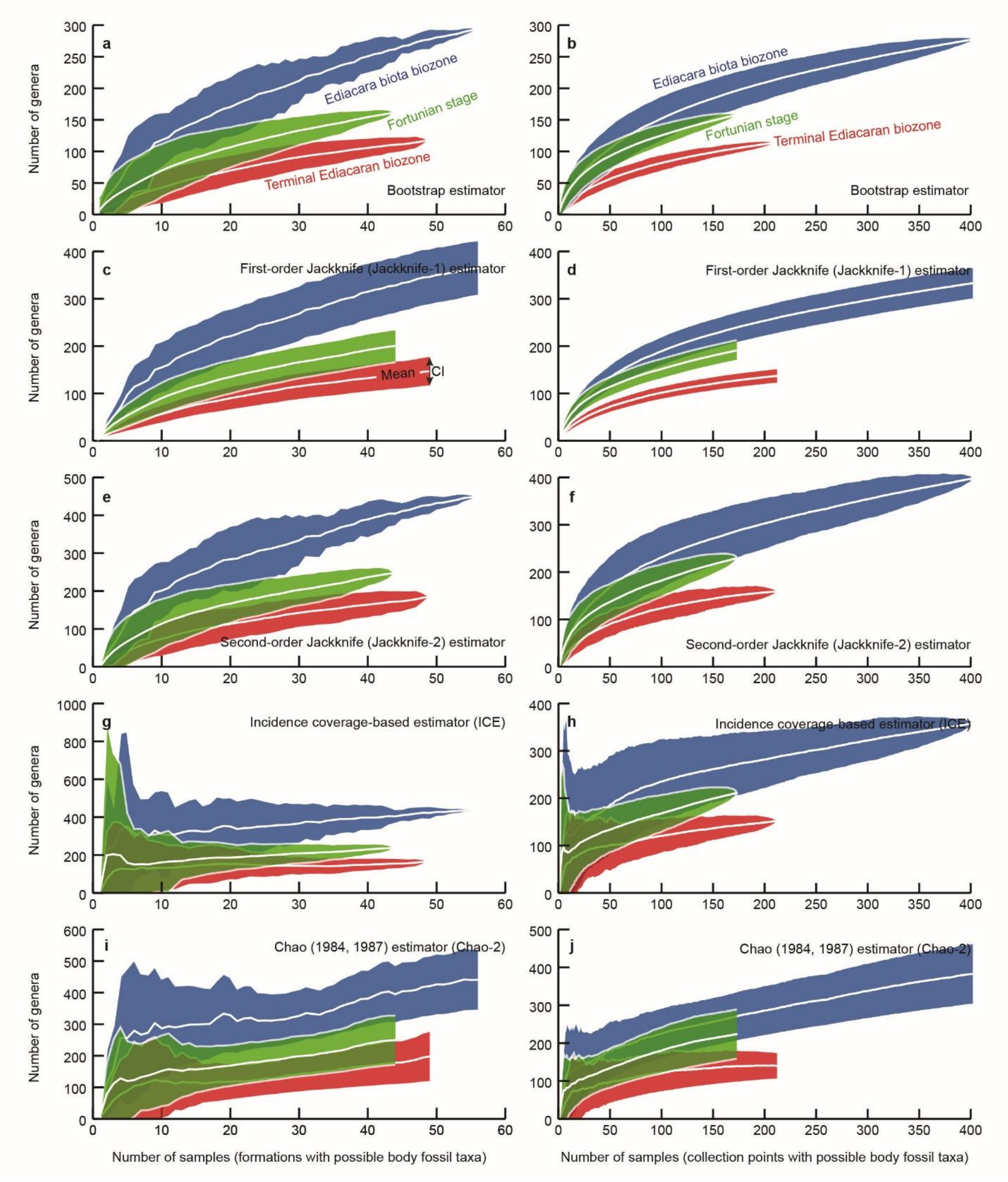

a
Ediacara biota biozone
Fortunian stage
Terminal Ediacaran biozone
Bootstrap estimator
b
Ediacara biota biozone
Fortunian stage
Terminal Ediacaran biozone
Bootstrap estimator
c
First-order Jackknife (Jackknife-1) estimator
Mean
CI
d
First-order Jackknife (Jackknife-1) estimator
e
Second-order Jackknife (Jackknife-2) estimator
f
Second-order Jackknife (Jackknife-2) estimator
g
Incidence coverage-based estimator (ICE)
h
Incidence coverage-based estimator (ICE)
i
Chao (1984, 1987) estimator (Chao-2)
j
Chao (1984, 1987) estimator (Chao-2)
Number of genera
Number of samples (formations with possible body fossil taxa)
Number of samples (collection points with possible body fossil taxa)

Supplementary Figure 15 | Generic diversity of biozones estimated using non-parametric richness estimators (all genera). Data includes all body fossil taxa, including discs and possible synonyms, taphomorphs, and microbially induced sedimentary structures. Plots show generic diversity (number of genera) versus sampling intensity for the stage-level biozones identified in this study. For a given sampling intensity level, plotted values of diversity are means of 1,000 randomizations of sample order and are bracketed by 95% confidence interval (CI) envelopes. The values were calculated using five different richness estimators[13]. (**a, b**) Bootstrap estimator. (**c, d**) First-order Jackknife (Jackknife-1) estimator. (**e, f**) Second-order Jackknife (Jackknife-2) estimator. (**g, h**) Incidence coverage-based estimator (ICE). (**i, j**) Chao (1984, 1987) estimator (Chao-2). The estimates were calculated from samples defined as geologic formations (**a, c, e, g, i**) and collection points (**b, d, f, h, j**) with relevant body fossils. Collection points were assigned to biozones based on the community assignments of their formations (Fig. 4; See Supplementary Figure 13). Whereas the CIs in (**a, b, e–h**) were calculated from conditional standard deviations determined via stochastic resampling, the CIs in (**c, d, i, j**) were calculated from unconditional standard deviations, which were derived using exact analytical methods[13]. For this reason, the unconditional confidence intervals in (**c, d, i, j**) do not converge to zero variance at the reference sample, like the conditional confidence intervals in (**a, b, e–h**). In general, non-overlap of 95% CIs constructed from unconditional variance estimators (Jackknife-1 and Chao-2) can be used as a simple but conservative criterion of statistical difference[13]. On the other hand, non-overlap of 95% CIs constructed from conditional variance estimators (Bootstrap, Jackknife-2 and ICE) can be used as a criterion that a smaller reference sample was not drawn from a larger one. Source data are provided as a Source Data file.

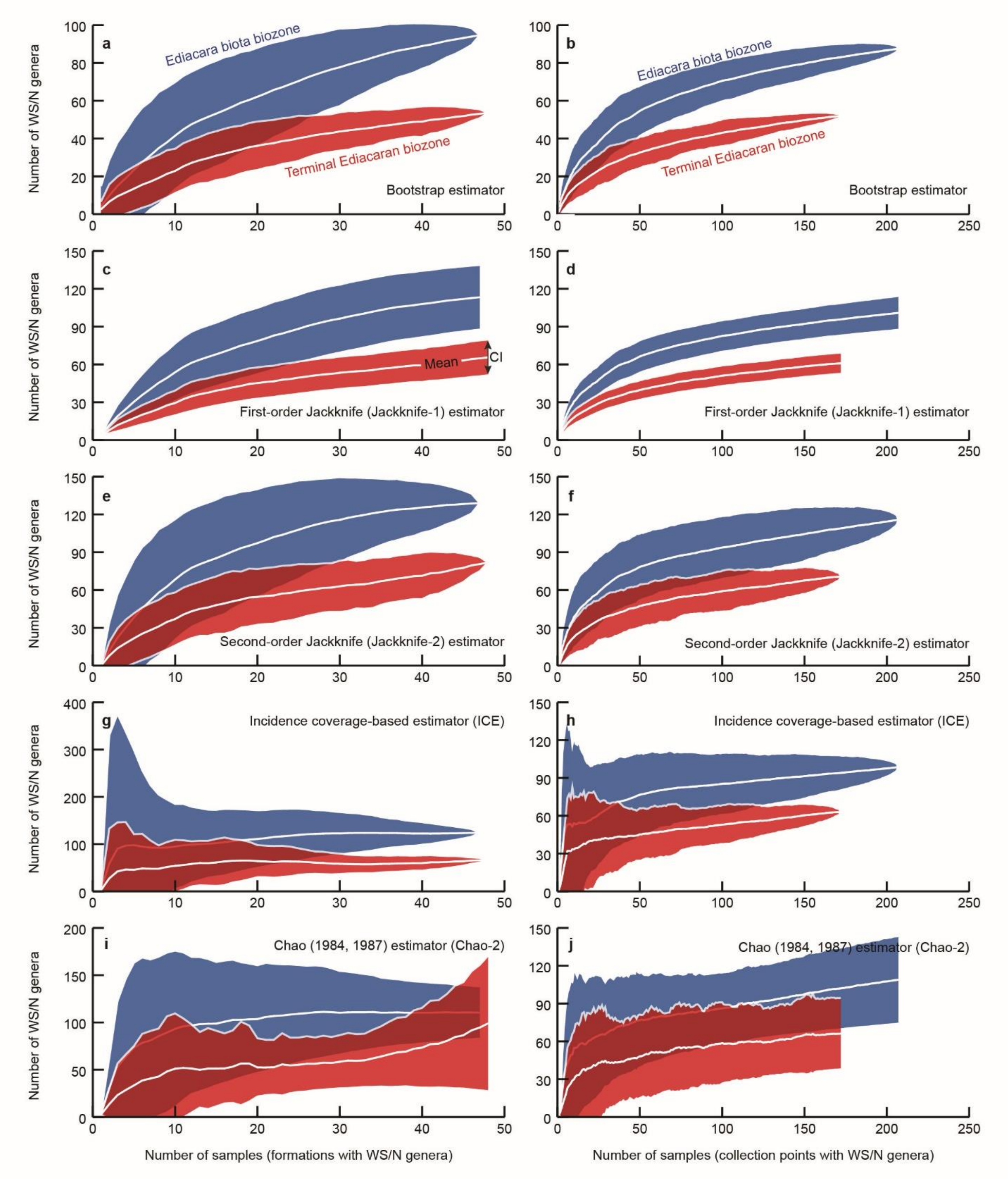
a
b
c
d
e
f
g
h
i
j
Ediacara biota biozone
Terminal Ediacaran biozone
Bootstrap estimator
First-order Jackknife (Jackknife-1) estimator
Second-order Jackknife (Jackknife-2) estimator
Incidence coverage-based estimator (ICE)
Chao (1984, 1987) estimator (Chao-2)
Mean
CI
Number of WS/N genera
Number of samples (formations with WS/N genera)
Number of samples (collection points with WS/N genera)

Supplementary Figure 16 | Generic diversity of biozones estimated using non-parametric richness estimators (all White Sea and Nama genera). Data includes all body fossil taxa assigned to the White Sea and/or Nama (WS/N) paleocommunities (Fig. 2a). Plots show generic diversity (number of genera) versus sampling intensity for the stage-level biozones identified in this study. For a given sampling intensity level, plotted values of diversity are means of 1,000 randomizations of sample order and are bracketed by 95% confidence interval (CI) envelopes. The values were calculated using five different richness estimators[13]. (**a, b**) Bootstrap estimator. (**c, d**) First-order Jackknife (Jackknife-1) estimator. (**e, f**) Second-order Jackknife (Jackknife-2) estimator. (**g, h**) Incidence coverage-based estimator (ICE). (**i, j**) Chao (1984, 1987) estimator (Chao-2). The estimates were calculated from samples defined as geologic formations (**a, c, e, g, i**) and collection points (**b, d, f, h, j**) with relevant body fossils. Collection points were assigned to biozones based on the community assignments of their formations (Fig. 4; See Supplementary Figure 13). Whereas the CIs in (**a, b, e–h**) were calculated from conditional standard deviations determined via stochastic resampling, the CIs in (**c, d, i, j**) were calculated from unconditional standard deviations, which were derived using exact analytical methods[13]. For this reason, the unconditional confidence intervals in (**c, d, i, j**) do not converge to zero variance at the reference sample, like the conditional confidence intervals in (**a, b, e–h**). In general, non-overlap of 95% CIs constructed from unconditional variance estimators (Jackknife-1 and Chao-2) can be used as a simple but conservative criterion of statistical difference[13]. On the other hand, non-overlap of 95% CIs constructed from conditional variance estimators (Bootstrap, Jackknife-2 and ICE) can be used as a criterion that a smaller reference sample was not drawn from a larger one. Source data are provided as a Source Data file.

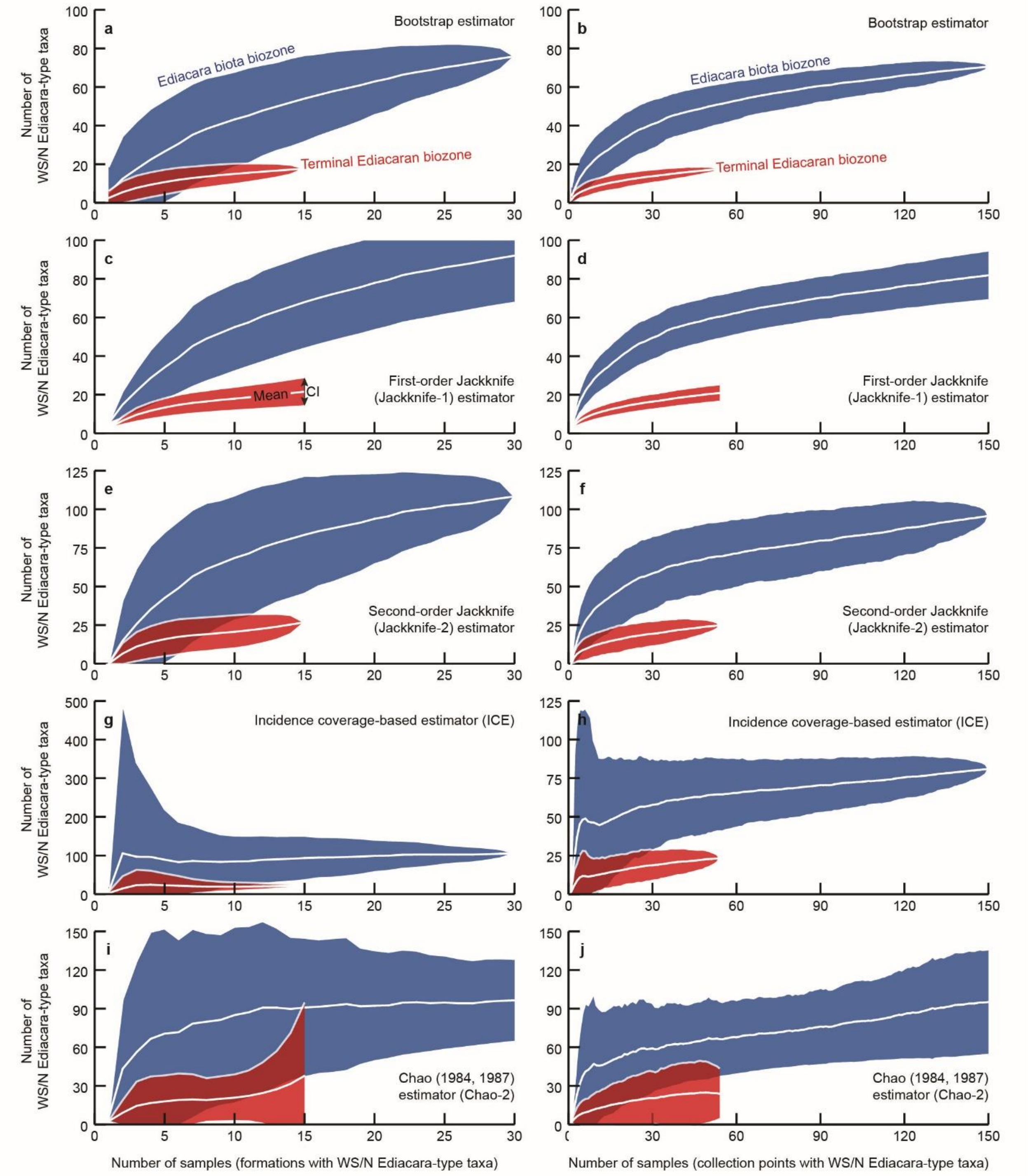

a
b
c
d
e
f
g
h
i
j
Bootstrap estimator
Ediacara biota biozone
Terminal Ediacaran biozone
First-order Jackknife
(Jackknife-1) estimator
Mean
CI
Second-order Jackknife
(Jackknife-2) estimator
Incidence coverage-based estimator (ICE)
Chao (1984, 1987)
estimator (Chao-2)
Number of
WS/N Ediacara-type taxa
Number of samples (formations with WS/N Ediacara-type taxa)
Number of samples (collection points with WS/N Ediacara-type taxa)

Supplementary Figure 17 | Generic diversity of biozones estimated using non-parametric richness estimators (White Sea and Nama Ediacara-type fossil taxa). Data includes all genera known from Ediacara-type fossils and assigned to the White Sea and/or Nama (WS/N) paleocommunities (Fig. 2a). Plots show generic diversity (number of genera) versus sampling intensity for the stage-level biozones identified in this study. For a given sampling intensity level, plotted values of diversity are means of 1,000 randomizations of sample order and are bracketed by 95% confidence interval (CI) envelopes. The values were calculated using five different richness estimators[13]. (**a, b**) Bootstrap estimator. (**c, d**) First-order Jackknife (Jackknife-1) estimator. (**e, f**) Second-order Jackknife (Jackknife-2) estimator. (**g, h**) Incidence coverage-based estimator (ICE). (**i, j**) Chao (1984, 1987) estimator (Chao-2). The estimates were calculated from samples defined as geologic formations (**a, c, e, g, i**) and collection points (**b, d, f, h, j**) with relevant body fossils. Collection points were assigned to biozones based on the community assignments of their formations (Fig. 4; See Supplementary Figure 13). Whereas the CIs in (**a, b, e–h**) were calculated from conditional standard deviations determined via stochastic resampling, the CIs in (**c, d, i, j**) were calculated from unconditional standard deviations, which were derived using exact analytical methods[13]. For this reason, the unconditional confidence intervals in (**c, d, i, j**) do not converge to zero variance at the reference sample, like the conditional confidence intervals in (**a, b, e–h**). In general, non-overlap of 95% CIs constructed from unconditional variance estimators (Jackknife-1 and Chao-2) can be used as a simple but conservative criterion of statistical difference[13]. On the other hand, non-overlap of 95% CIs constructed from conditional variance estimators (Bootstrap, Jackknife-2 and ICE) can be used as a criterion that a smaller reference sample was not drawn from a larger one. Source data are provided as a Source Data file.

Supplementary Table 1 | Glossary of network analysis terms.

Assemblage	A term for any associated group of specimens, localities, or samples. For instance, it may describe the animal fossils found in a given geologic stratum, either locally at a collection point or globally within a biozone; localities that are taxonomically similar to one another; or samples thought to represent a single population.
Assortativity	A score of homophily (see also: homophily).
Average degree	The mean degree score calculated from the nodes in one or more assemblages (communities, modules, biotopes, or biozones) in a network (see also: degree).
Betweenness centrality	The number of shortest paths that pass through a node in a network.
Biotope (network-based)	A distinctive association of interacting taxa and their physical environment, which occupy a continuous geographical area that can be delimited by convenient boundaries. Defining environmental features generally include a substrate, hydrodynamic conditions, and light. In network analysis, it is defined as an association (community or module) made of taxa and environment nodes.
Biozone (network-based)	Biostratigraphic zone. An interval of geological strata, which is defined and can be recognized interregionally through stratigraphic correlation on the basis of its fossil content (i.e. taxa). In networks analysis, it is defined as an association (community or module) made of taxa and geologic unit nodes. This association most closely resembles an assemblage biozone, defined by a unique association of three or more taxa, which distinguish it from adjacent strata.
Bipartite network	A network containing two types of nodes. Nodes of the first type are always connected to nodes of the second, and vice versa, but nodes of the same type are never connected to each other (see also: projection).
Centrality	The importance, however defined, of a node within a network. Centrality scores typically measure (1) the relative roles that nodes play in the flow of information through their networks (e.g. how often they serve as a bridge or how well connected they are to each other) and (2) the closeness of the nodes to the others.
Clique	A subset of nodes/vertices, in which, every node in the subset is connected to every other. The most basic level of organization in an undirected multi-modal structure.
Cluster	This term is used to describe the outputs of a number of statistical and analytical methods, including hierarchical clustering and network partitioning. In general, it refers to any group of units (e.g. individuals, taxa, or samples), which are more closely associated (related or similar) to each other than the others in the data.
Community (network-based)	Equivalent to a network module (see also: module). A cluster of nodes within a network, usually identified via statistical and analytical means, representing closely associated entities. Depending on the type(s) of its nodes, it may or may not represent an ecological/biological community or paleocommunity.
Community structure (network partition)	The output of a community algorithm, consisting of multiple communities/modules/clusters of nodes.
Community-detection algorithm	A function that identifies the community structure of a network on the basis of some definition of community. The computational method generally involves partitioning a network by assigning its nodes to overlapping or non-overlapping modules/communities of number (n).
COPRA algorithm	A label propagation method of network partitioning for identifying overlapping and non-overlapping communities within unipartite and bipartite networks.
Degree (degree centrality)	The number of connections incident on a node/vertex within a given network. The number of links between a node and any other, including itself, if self-loops are allowed (see also: degree centrality and self-loops).
Degree distribution	The distribution of degree/degree centrality values in a network.
Diameter	The number of connections/links/edges separating the most distant vertices in a network.
Directed network	A network, in which connections/links/edges have orientations and directionality (e.g. a connection goes from node A to node B, but not from node B to node A).

Supplementary Table 1 (cont.) | Glossary of network analysis terms.

Edge	A connection between a pair of nodes/vertexes in a network, representing an interaction between those entities (see also: link).
Edge Density	The ratio between the number of actual connections/links/edges and the number of possible connections/links/edges within a network.
Edge-betweenness algorithm	A function for community detection that seeks to identify community structure in a network by partitioning communities at the connections/links/edges that are most commonly found in the shortest paths between nodes
Fast greedy algorithm	A function for network partitioning and community detection that seeks to identify the best community structure of a network by optimizing its modularity.
Fruchterman-Reingold force-directed algorithm	A force-directed algorithm for defining relative node positions and connection lengths in a network graph.
Global network property	An emergent attribute of a whole network, which can usually be described with a single value or score.
Homophily	The tendency of entities to associate with others possessing similar properties.
Infomap algorithm	A function for network partitioning and community detection that seeks to identify community structure in a network by minimizing the expected description length of a random walker trajectory.
Jackknife network	A network produced by jackknife resampling of the nodes and links in a parent network, such that, each node is left out of exactly one jackknife network and a total number of jackknife networks is produced that is equal to the size of the parent network. These jackknife networks are used for estimating the *v* parameter used in network partitioning with the COPRA algorithm (see also: jackknife resampling, *v* parameter, and COPRA algorithm).
Label propagation method	Any community detection algorithm/network partitioning method that involves randomly assigning unique labels to the nodes in a network in an initial step and repeatedly revising those labels, so that over multiple iterations, the nodes take on the most common labels among their direct neighbors, leading to discovery of densely connected groups of nodes (i.e. communities). Because this approach involves randomly assigning labels to nodes at the start, it may produce any number of outputs, rather than a single optimal community structure. For this reason, label propagation methods must be repeated numerous times to ensure that the best community structure has been found (see also: COPRA algorithm).
Leading eigenvector algorithm	A function for network partitioning and community detection that seeks to identify the best community structure of a network by optimizing its modularity.
Link	A connection between a pair of nodes/vertexes in a network, representing an interaction between those entities (see also: edge)
Local network property	An attribute of a specific node/vertex within a network, which can usually be described with a single value or score.
Louvain (multi-level) algorithm	A function for network partitioning and community detection that seeks to identify the best community structure of a network by optimizing its modularity.
Maximum degree	The highest degree score in one or more assemblages (e.g. communities, modules, biotopes, or biozones) in a network (see also: degree).
Mixing parameter	An attribute of a community that specifies its level of connection with others
Modularity (Q)	A measure of the strength of division that ranges between 0 and 1 and provides an indication of community structure within a network (see also: main text).
Module	Equivalent to a network community. A cluster of nodes within a network, usually identified via statistical and analytical means, representing closely associated entities (see also: community).

Supplementary Table 1 (cont.) | Glossary of network analysis terms.

Multiple edges (multiple links)	Two or more connections that are incident upon the same two nodes/vertices in a network (see also: edges and links). These types of connections are not present in simple networks and graphs.
Multipartite network	A network containing two or more types of nodes/vertices, and two or more projections. A bipartite network, which contains two types of node/vertices and two projections, is an example of a multipartite network.
Multi-modal data structure	A pseudo-hierarchical, nested data structure with levels of organization: microscale cliques, mesoscale communities, and macroscale clusters of communities.
Network	A group of independent but associated entities (see also community)
Network size	The total number of nodes/vertices within a network.
Node	An entity within a network (see also: vertex)
Non-overlapping communities	Communities that do not share any element (nodes) in common. Two or more modules that are mutually exclusive with respect to one another.
Non-weighted (unweighted) network	A network containing connections/links/edges of uniform strength. In these networks, there are no differences in the magnitudes of connection among pairs of nodes.
Overlapping communities	Communities that share any number of elements (nodes) in common. Two or more modules that are not mutually exclusive with respect to one another.
Paired singleton community	A community/module in a bipartite network, such that, one of the projections in the community/module is represented by a single node
Projection	A subset of nodes in a network, which typically represent a common type. In bipartite networks, there are two projections, one for each type of node. These projections can be derived from bipartite networks by compressing the data and wiring nodes to neighbors of their neighbors. Accordingly, the projections can be visualized and analyzed as separate and independent unipartite networks.
Self-loop (or loop)	A connection between a node/vertex and itself. Not present in simple networks.
Simple network	A network or graph without multiple edges or self-loops.
Singleton community	A community in a unipartite network that contains exactly one node.
Transitivity	A coefficient measuring the probability that adjacent vertices of a node are connected to each other.
Undirected network	A network, in which connections/links/edges do not have orientations or directionality (e.g. a connection goes from node A to node B, and vice versa),
Unipartite network	A network with one type of node. Any pair of nodes may be connected, and the data does not support additional projections.
v parameter	The maximum number of communities/modules that a node may belong in a network with overlapping community structure. A parameter in the COPRA algorithm for network partitioning and community detection.
Vertex	An entity within a network (see also: node)
Walktrap algorithm	A function for network partitioning that seeks to identify the best community structure of a network with short random walks that tend to stay within their own communities.
Weighted network	A network containing connections/links/edges of unequal strength. In these networks, nodes in some pairs are more strongly connected than nodes in other pairs.

Supplementary Table 2 | Preservational modes of Ediacaran macrofossils and taxa.

Mode	Description
Carbonaceous compressions	Taxa known exclusively from carbonaceous compressions, which exhibit minor or no (non-replacive and non-pervasive) mineralization.
Ediacara-type fossils	Taxa known exclusively from casts, moulds, and impressions in siliciclastic rocks and/or limestone; fossils in limestone may additionally reflect mineralization via calcification.
Mineralized fossils	Taxa known exclusively from mineralized fossils (i.e. fossils with evidence of pervasive pyritization, silicification, phosphatization, and/or aluminosilicification).
Skeletal fossils	Taxa known from fossils of agglutinated and/or biomineralized skeletons, which may be preserved as steinkerns, external moulds, or secondary authigenic/diagenetic minerals.
Trace fossils	Ichnotaxa known from siliciclastic and carbonate rocks. This category includes predation traces.
Carbonaceous compressions & mineralized fossils	Taxa known exclusively from carbonaceous compressions, mineralized fossils, and fossils preserved with both organic and authigenic/diagenetic minerals.
Ediacara-type fossils & carbonaceous compressions	Taxa known from Ediacara-type fossils and carbonaceous compressions. Examples include *Eoandromeda*, *Gesinella*, *Flabelophyton*, *Liulingjitaenia*, and *Longifuniculum*, which are preserved as carbonaceous compressions in the Doushantuo Formation but Ediacara-type fossils in the Rawnsely Quartzite.
Other or unknown modes of preservation	Taxa do not match the criteria of any of the other categories, or there is insufficient data for categorization. These taxa include *Kelleria* (known from a single specimen, which is now missing) as well as *Horodyskia*, *Palaeopaschichnus*, and *Yelovichnus*, which are potentially known from Ediacara-type fossils, mineralized fossils, and carbonaceous compressions.

Supplementary Table 3 | Trace architecture designs and architectural form categories.

General descriptor (Form category)	Trace-fossil architectural design (Morphogroup)	Interpretation
Scratch marks	Scratch mark	Feeding trace of mat grazers and detritus feeder
Skeletal fossil borings	Circular holes (borings) in skeletal fossil	Feeding trace (predation mark) of an active predator
Impressions	Oval-shaped impression	Resting trace of mat digester
	Bilaterally symmetrical short, shallow to deep scratched impressions	Resting trace of deposit and detritus feeder
	Pentameral-shaped impression or burrow	Resting trace of passive predators or detritus feeder
Trackways	Simple horizontal trail	Grazing trail of deposit and detritus feeder (including mat grazers)
	Actively filled (complex meniscate) horizontal burrow	Feeding structure of deposit feeder
	Circular trail	Feeding structure of deposit feeder
	Bilobate trail with paired grooves	Locomotion trace of deposit and detritus feeder
	Trilobate flattened trail	Locomotion trace of an active predator
	Trackway	Locomotion trace of deposit and detritus feeder
Burrows	Horizontal branched burrow system	Feeding structure of deposit feeder (possibly an undermat miner)
	Actively filled (massive) horizontal burrow	Feeding structure of deposit feeder
	Horizontal to oblique branching burrow	Feeding structure of deposit and detritus feeder (possibly undermat miner)
	Passively filled horizontal burrow	Dwelling burrow of suspension feeder and/or active predator
	Plug-shaped burrow	Dwelling burrow of suspension feeder and/or passive predator
	Vertical helicoidal burrow	Feeding burrow of deposit feeder
	Surface-coverage branching burrow	Feeding structure of undermat miner
	Radial branching ichnological structure	Feeding structure of deposit feeder
	Burrow with vertical spreiten	Feeding structure of deposit feeder
	Burrow with horizontal spreiten	Feeding structure of deposit feeder
	Mazes and boxworks (i.e. galleries)	Feeding structure of deposit feeder
Graphoglyptids	Radial graphoglyptid	Farming trace
	Regular network graphoglyptid	Farming trace

Supplementary Table 4 | Paleoenvironments used in this study.

Paleoenvironment	Definition	General description of facies
Fluvial setting	A riverine environment.	Channelized, cross-stratified, medium- to coarse-grained sandstone and conglomerate with evidence of unidirectional flow
Subtidal lagoon setting	A restricted setting (e.g. intrashelf basin or cove) with quiet hydrodynamic conditions.	Structureless, thinly laminated shale, mudstone, siltstone, and/or wackestone.
Supratidal zone / backshore (Clastic)	The area from the fair-weather high tide mark to the extreme limit of a beach, which is inundated during storms.	Well-sorted and well-rounded, fine- to medium-grained sandstone with large-scale low-angle cross-bedding.
Intertidal zone / foreshore (Clastic)	The area under water at high tide and above water at low tide	Sandstone with primary current lineation (i.e. seaward-dipping planar cross-bedded laminae), rill marks, and adhesion ripples.
Upper shoreface / Proximal delta front (“mouth bar”) (Clastic)	The area between the low tide line, and wave break marks, where sediments are subjected to multidirectional current flows in the build-up and surf zones (plus fluvial discharge in the case of delta front).	Fine- to medium-grained sandstone with asymmetrical ripples and dunes and planar laminated and trough cross-stratified beds
Lower shoreface / Distal delta front (Clastic)	The area below the wave break mark and immediately above the fair-weather wave base, where sediments are dominantly affected by wave action (plus fluvial discharge in the case of delta front).	Contorted and hummocky cross-stratified, erosive-based, fine- to very fine-grained sandstone. Symmetrical ripples and parallel lamination are present.
Offshore shelf transition / Prodelta (Clastic)	The area between fair-weather and storm wave bases, where sediments are sometimes affected by wave action (plus fluvial discharge in the case of prodelta).	Fine- to very fine-grained sandstone, siltstone, and mudstone. Ripples and hummocky cross-stratified beds are present.
Offshore shelf (Clastic)	The area below storm wave base on a shelf dominated by suspension fallout deposition.	Structureless mudstone and siltstone with thin, laminated, graded beds of fine-grained sandstone. Thin- to medium-bedded storm-generated turbidites are present as sole-marked sandstones with parallel lamination.
Slope & basin (Clastic)	The area off the shelf break dominated by gravity flow and hemipelagic deposition.	Shale and mudstone containing sandstone turbidites, slumps, and exotic blocks.
Supratidal zone / backshore (Carbonate)	The area from the fair-weather high tide mark to the extreme limit of a beach, which is inundated during storms.	Dolostone with small-scale cross beds and desiccation structures.
Intertidal zone / foreshore (Carbonate)	The area under water at high tide and above water at low tide.	Algal laminated limestone and dolostone with desiccation structures, tidal channels, and small-scale cross beds.
Reef (Carbonate)	A biologically mediated carbonate build-up or bioherm of metazoans or microbes.	Limestone and dolostone with grainstone, packstone, stromatolites, thrombolites.
Inner ramp (Carbonate)	The area below the low tide mark and above fairweather wave base.	Grainstone and packstone with oolitic ridges interrupted by clastic intervals. Carbonates and clastics are segregated.
Middle ramp (Carbonate)	The area below fairweather wave base and storm wave base.	Wackestone and mudstone. Carbonates and clastics (shale and siltstone) are well mixed.
Outer ramp (Carbonate)	The area on a carbonate shelf below storm wave base.	Micritic limestone with calcisiltite, shale, siltstone, and sandstone. Carbonates and clastics are well mixed.
Shelf edge barrier (Carbonate)	The area on a carbonate shelf between a lagoon/basin and the slope/basin, where sediment occurs relatively shallow depths.	Oolitic lime sandstone or dolostone with grainstone and evidence of exposure. Carbonates and clastics are well mixed.
Slope & basin (Carbonate	The area off a ramp or shelf dominated by deposition of carbonate rock.	Shale and mudstone with slump features. Carbonates and clastics are segregated.

Supplementary Table 5 | Non-metric multidimensional scaling scores. Scores were calculated from Kulczynski-2 and Jaccard indexes of taxonomic dissimilarity for 34 geologic formations of Ediacaran age containing 5 or more macrofossil genera or ichnogenera. Three scores were calculated for each formation and index.

	Kulczynski-2 index			Jaccard index		
**Geologic formation**	**NMDS1**	**NMDS2**	**NMDS3**	**NMDS1**	**NMDS2**	**NMDS3**
"Ust-Pinega formation" (Russia)	-1.26	-0.16	-0.80	-1.26	-0.16	-0.63
Basa Formation (Russia)	0.03	0.43	0.52	0.03	0.51	0.47
Blueflower Formation (Canada)	0.39	0.55	-0.19	0.39	0.44	-0.21
Bradgate Formation (UK)	-0.91	0.95	0.35	-0.79	0.85	0.18
Briscal Formation (Canada)	-1.37	0.78	0.16	-1.28	0.77	0.20
Chernyi Kamen Formation (Russia)	-0.48	-0.32	-0.03	-0.58	-0.19	0.04
Dabis Formation (Namibia)	0.93	-0.15	-0.20	0.79	-0.13	-0.19
Deep Spring Formation (USA)	1.13	0.90	0.05	1.32	0.64	-0.21
Dengying Formation (China)	0.74	0.46	0.12	0.74	0.41	0.05
Doushantuo Formation (China)	0.13	-1.62	0.46	0.02	-1.46	0.53
Drook Formation (Canada)	-0.90	0.91	-0.13	-0.81	0.89	-0.17
Erga Formation (Russia)	-0.18	-0.54	-0.37	-0.14	-0.53	-0.45
Fermeuse Formation (Canada)	-0.91	0.52	0.34	-0.74	0.59	0.27
Floyd Church Formation (USA)	0.74	0.39	-0.93	0.86	0.29	-0.93
Kauriyala Formation (India)	-0.21	0.21	-0.76	-0.21	0.31	-0.75
Khatyspyt Formation (Russia)	-0.18	-0.44	0.94	-0.17	-0.28	0.86
Lantian Formation (China)	-0.26	-2.47	-0.77	-0.64	-2.25	-0.74
Lyamsta Formation (Russia)	-0.55	-0.97	0.53	-0.55	-0.75	0.60
Mistaken Point Formation (Canada)	-0.97	1.00	0.12	-0.84	0.94	0.00
Mogilev Formation (Ukraine)	-0.34	-0.42	-0.89	-0.40	-0.34	-0.63
Nadaleen Formation (Canada)	-0.88	0.83	0.02	-0.76	0.85	-0.10
Nagoryany Formation (Ukraine & Moldova)	1.01	-0.58	1.09	0.84	-0.58	1.07
Nudaus Formation (Namibia)	0.70	-0.07	-0.71	0.80	-0.10	-0.83
Perevalok Formation (Russia)	-0.40	-0.95	0.93	-0.54	-0.57	1.02
Raiga Formation (Russia)	1.59	0.07	0.64	1.44	-0.14	0.66
Rawnsley Quartzite Formation (Australia)	-0.12	0.00	-0.52	-0.06	-0.14	-0.57
Studenitsa Formation (Ukraine & Moldova)	0.33	-0.15	1.01	0.23	0.00	0.91
Trepassey Formation (Canada)	-0.97	0.95	0.14	-0.85	0.86	0.04
Urusis Formation (Namibia)	1.21	0.21	-0.12	1.10	0.08	-0.07
Verhovka Formation (Russia)	-0.28	-0.46	-0.48	-0.17	-0.68	-0.38
Wood Canyon Formation (USA)	1.78	0.49	-0.31	1.56	0.51	0.17
Yaryshev Formation (Ukraine)	-0.85	-0.32	0.51	-0.72	-0.14	0.49
Zaris Formation (Namibia)	1.43	0.73	-0.20	1.62	0.22	-0.27
Zimnie Gory Formation (Russia)	-0.15	-0.76	-0.52	-0.23	-0.74	-0.44

Supplementary Table 6 | Results of various network partitioning methods (See Supplementary Figure 5). Results include the number of non-overlapping communities (n) and the associated extended modularity score (Q). Results are included for two versions of the unipartite network of Ediacaran genera: one with weighted and one with non-weighted links. Values are provided for all of the unipartite projections of the bipartite networks in this study.

Network	Algorithm	Nodes	n	Q	Nodes	n	Q
**Unipartite network of Ediacaran genera**	**COPRA**	**Genera (nonweighted links)**	4	0.84	**Genera (weighted links)**	4	0.81
	**Leading eigenvector**		4	0.83		7	0.83
	**Louvain**		6	0.81		5	0.78
	**Fast Greedy**		5	0.74		4	0.80
	**Infomap**		9	0.81		9	0.76
	**Walktrap**		9	0.81		16	0.75
	**Edge-betweenness**		23	0.80		6	0.82
**Bipartite network of paleoenvironments and Ediacaran genera**	**COPRA**	**Environments**	3	0.39	**Genera**	3	0.52
	**DIRTLPAwb+**		7	0.14		7	0.35
	**LPAwb+**		13	0		13	0.23
	**LPBRIM**		7	0.16		7	0.36
	**QuanBiMo**		2	0.14		2	0.04
	**Simulated annealing**		3	0.29		11	0.59
	**Adaptive BRIM**		4	0.23		4	0.49
	**Leading eigenvector**		6	0.14		6	0.41
	**biSBM**		4	0.21		4	0.24
**Bipartite network of paleoenvironments and Ediacaran genera & ichnogenera**	**COPRA**	**Environments**	2	0.42	**Genera & ichnogenera**	2	0.47
	**DIRTLPAwb+**		6	0.21		6	0.39
	**LPAwb+**		13	0.00		13	0.21
	**LPBRIM**		6	0.19		6	0.39
	**QuanBiMo**		2	0.15		2	0.09
	**Simulated annealing**		3	0.34		11	0.57
	**Adaptive BRIM**		4	0.21		4	0.46
	**Leading eigenvector**		6	0.12		6	0.40
	**biSBM**		4	0.16		4	0.21
**Bipartite network of Ediacaran formations and genera & ichnogenera**	**COPRA**	**Formations**	6	0.64	**Genera & ichnogenera**	6	0.55
	**DIRTLPAwb+**		6	0.44		6	0.52
	**LPAwb+**		55	0.10		55	0.25
	**LPBRIM**		17	0.39		17	0.52
	**QuanBiMo**		6	0.14		6	0.17
	**Simulated annealing**		11	0.56		13	0.63
	**Adaptive BRIM**		6	0.44		6	0.60
	**Leading eigenvector**		8	0.45		8	0.55
	**biSBM**		4	0.32		4	0.27
**Bipartite network of Ediacaran formations and shallow genera**	**COPRA**	**Formations**	4	0.78	**Genera**	4	0.67
	**DIRTLPAwb+**		9	0.38		9	0.47
	**LPAwb+**		33	0.10		33	0.23
	**LPBRIM**		11	0.41		11	0.47
	**QuanBiMo**		11	0.30		11	0.39
	**Simulated annealing**		9	0.72		8	0.67
	**Adaptive BRIM**		6	0.47		6	0.52
	**Leading eigenvector**		9	0.38		9	0.52
	**biSBM**		4	0.36		2	0.27
**Bipartite network of Ediacaran formations and shallow genera & ichnogenera**	**COPRA**	**Formations**	3	0.73	**Genera & ichnogenera**	3	0.65
	**DIRTLPAwb+**		8	0.39		8	0.39
	**LPAwb+**		41	0.11		41	0.24
	**LPBRIM**		11	0.38		11	0.47
	**QuanBiMo**		11	0.33		11	0.37
	**Simulated annealing**		13	0.67		9	0.62
	**Adaptive BRIM**		6	0.35		6	0.53
	**Leading eigenvector**		9	0.29		9	0.49
	**biSBM**		4	0.33		2	0.27

Supplementary Table 7 | Whole-network properties. Each row is a taxa projection, paleoenvironments projection (Environs.), or formations projection (Fms.) of a network in this study. Diameter (D), transitivity (T), modularity (Q), and edge-density (E-D) metrics were calculated for each projection. Diameter is the maximum degree of separation; transitivity is a measure of triadic closure (i.e. the probability that neighbors of a node are connected); and edge density is the ratio between the numbers of actual connections and possible links within a given network.

Network	Projection	D	Q	T	E-D	Figure
Unipartite (genera)	Taxa	6	0.82	0.59	0.09	Fig. 2a
Bipartite (paleoenvironments/ Ediacaran genera)	Environs.	2	0.39	0.83	0.78	Fig. 3a
	Taxa	3	0.51	0.79	0.41	
Bipartite (paleoenvironments/Ediacaran genera & ichnogenera)	Environs.	2	0.38	0.85	0.82	Supplementary Fig. 10a
	Taxa	3	0.50	0.80	0.44	
Bipartite (Ediacaran formations/ genera & ichnogenera)	Fms.	4	0.63	0.58	0.21	Fig. 4a
	Taxa	5	0.51	0.62	0.16	
Bipartite (Ediacaran formations/shallow biotope genera & ichnogenera)	Fms.	5	0.69	0.62	0.22	Supplementary Fig. 11a
	Taxa	5	0.65	0.69	0.30	
Bipartite (Ediacaran formations/ shallow biotope genera)	Fms.	6	0.74	0.72	0.23	Supplementary Fig. 12a
	Taxa	6	0.66	0.73	0.28	
Bipartite (Ediacaran-Fortunian formations/ genera & ichnogenera)	Fms.	5	0.64	0.60	0.15	Supplementary Fig. 13a
	Taxa	6	0.77	0.62	0.10	

Supplementary Table 8 | Assortativity coefficients. Each row is a taxa projection or formations projection (Fms.) of a network in this study. Assortativity coefficients measure homophily (mixing) of nodes with respect to nominal and continuous properties. Assortativity coefficients, measuring mixing with respect to node degrees (D), were determined for all projections. Additionally, assortativity coefficients were calculated for taxa projections from data on the preservational modes (P), morphogroups (M), and form categories (F) of the genera and ichnogenera. Lastly, assortativity coefficients were calculated for formation projections from data on the geoplates (G), regions/continents (R), and countries (C) of the geologic units.

Network	Projection	D	P	M	F	G	R	C	Figure
Unipartite (genera)	Taxa	-0.05	0.49	0.11	0.24	N/A	N/A	N/A	Fig. 2a
Bipartite (paleoenvironments/ Ediacaran genera)	Taxa	0.09	0.15	0.02	0.04	N/A	N/A	N/A	Fig. 3a
Bipartite (paleoenvironments/Ediacaran genera & ichnogenera)	Taxa	0.10	0.11	0.01	0.03	N/A	N/A	N/A	Suppl. Fig. 10a
Bipartite (Ediacaran formations/ genera & ichnogenera)	Fms.	-0.14	N/A	N/A	N/A	0.03	0.08	0.07	Fig. 4a
	Taxa	0.00	0.29	0.06	0.12	N/A	N/A	N/A	
Bipartite (Ediacaran formations/shallow biotope genera & ichnogenera)	Fms.	-0.14	N/A	N/A	N/A	0.03	0.04	0.06	Suppl. Fig. 11a
	Taxa	-0.07	0.12	0.03	0.09	N/A	N/A	N/A	
Bipartite (Ediacaran formations/ shallow biotope genera)	Fms.	-0.05	N/A	N/A	N/A	0.03	0.04	0.08	Suppl. Fig. 12a
	Taxa	0.01	0.17	0.04	0.12	N/A	N/A	N/A	
Bipartite (Ediacaran-Fortunian formations/ genera & ichnogenera)	Fms.	0.01	N/A	N/A	N/A	0.06	0.10	0.09	Suppl. Fig. 13a
	Taxa	0.01	0.62	0.12	0.40	N/A	N/A	N/A	

SUPPLEMENTARY REFERENCES

1 Gregory, S. Finding overlapping communities in networks by label propagation. *New J. Phys.* **12**, 1–21 (2010).

2 Raghavan, U. N., Albert, R. & Kumara, S. Near linear time algorithm to detect community structures in large-scale networks. *Phys. Rev. E* **76**, 1–12 (2007).

3 Antcliffe, J. B., Callow, R. H. T. & Brasier, M. D. Giving the early fossil record of sponges a squeeze. *Biol. Rev.* **89**, 972–1004 (2014).

4 Muscente, A. D., Michel, F. M., Dale, J. G. & Xiao, S. Assessing the veracity of Precambrian 'sponge' fossils using *in situ* nanoscale analytical techniques. *Precambrian Res.* **263**, 142–156 (2015).

5 Oksanen, J. F. *et al.* Vegan: Community Ecology Package. *R package version 2.0-10. http://CRAN.R-project.org/package=vegan*. (2015).

6 Milligan, G. W. & Cooper, M. C. Methodology review: Clustering methods. *Appl. Psychol. Meas.* **11**, 329–354 (1987).

7 Csardi, G. & Nepusz, T. The igraph software package for complex network research. *InterJournal Complex Systems* **1695**, 1–9 (2006).

8 Dormann, C. F., Fründ, J., Blüthgen, N. & Gruber, B. Indices, graphs and null models: Analyzing bipartite ecological networks. *The Open Ecology Journal* **11**, 7–24 (2009).

9 Flores, C. O., Poisot, T., Valverde, S. & Weitz, J. S. BiMat: a MATLAB package to facilitate the analysis of bipartite networks. *Methods Ecol. Evol.* **7**, 127–132 (2016).

10 Larremore, D. B., Clauset, A. & Jacobs, A. Z. Efficiently inferring community structure in bipartite networks. *Phys. Rev. E* **90**, 1–12 (2014).

11 Nicosia, V., Mangioni, G., Carchiolo, V. & Malgeri, M. Extending the definition of modularity to directed graphs with overlapping communities. *J. Stat. Mech. Theory E.* **2009**, 1–22 (2009).

12 Karrer, B., Levina, E. & Newman, M. E. J. Robustness of community structure in networks. *Phys. Rev. E* **77**, 1–10 (2008).

13 Colwell, R. K. EstimateS: Statistical estimation of species richness and shared species from samples. Version 9. User's Guide and application. http://purl.oclc.org/estimates (2006).
