# Supplementary material for: Ediacaran biozones identified with network analysis provide evidence for pulsed extinctions of early complex life
Source: Nat Commun. 2019 Feb 22;10:911. doi: 10.1038/s41467-019-08837-3 (PMC6384941; doi:10.1038/s41467-019-08837-3)
Supplement: Supplementary file 3 — Reporting Summary [file 41467_2019_8837_MOESM3_ESM.pdf]

nature research

Corresponding author(s): A. D. Muscente

Last updated by author(s): 2019-1-9

Reporting Summary

Nature Research wishes to improve the reproducibility of the work that we publish. This form provides structure for consistency and transparency in reporting. For further information on Nature Research policies, see Authors & Referees and the Editorial Policy Checklist.

Statistics

For all statistical analyses, confirm that the following items are present in the figure legend, table legend, main text, or Methods section.

n/a	Confirmed	
☐	☒	The exact sample size (*n*) for each experimental group/condition, given as a discrete number and unit of measurement
☐	☒	A statement on whether measurements were taken from distinct samples or whether the same sample was measured repeatedly
☐	☒	The statistical test(s) used AND whether they are one- or two-sided<br>*Only common tests should be described solely by name; describe more complex techniques in the Methods section.*
☐	☒	A description of all covariates tested
☐	☒	A description of any assumptions or corrections, such as tests of normality and adjustment for multiple comparisons
☐	☒	A full description of the statistical parameters including central tendency (e.g. means) or other basic estimates (e.g. regression coefficient) AND variation (e.g. standard deviation) or associated estimates of uncertainty (e.g. confidence intervals)
☐	☒	For null hypothesis testing, the test statistic (e.g. *F*, *t*, *r*) with confidence intervals, effect sizes, degrees of freedom and *P* value noted<br>*Give P values as exact values whenever suitable.*
☒	☐	For Bayesian analysis, information on the choice of priors and Markov chain Monte Carlo settings
☒	☐	For hierarchical and complex designs, identification of the appropriate level for tests and full reporting of outcomes
☒	☐	Estimates of effect sizes (e.g. Cohen's *d*, Pearson's *r*), indicating how they were calculated

Our web collection on statistics for biologists contains articles on many of the points above.

Software and code

Policy information about availability of computer code

Data collection	No software was used in data collection
Data analysis	Hierarchical clustering analyses were performed in RStudio using functions of the open source vegan (version 2.5.2) and pvclust (version 2.0.0) packages.<br><br>Multidimensional scaling analyses were performed in RStudio using functions of the open source vegan (version 2.5.2) and vegan3D (version 1.1.1) packages.<br><br>Network were analyzed in RStudio using functions in the following open source packages: igraph (version 1.2.2), BiRewire (version 3.12.0), bipartite (version 2.11.0), lpbrim (version 1.0.0), and rnetcarto (version 0.2.4). Networks were also analyzed in MATLAB using the BiMat package, in C++ using the code made available by D. Larremore (http://danlarremore.com/bipartiteSBM/), and in JAVA using the COPRA program produced by S. Gregory (http://gregory.org/research/networks/software/copra.html).<br><br>Networks were visualized in RStudio using functions in the following open source packages: igraph (version 1.2.2), GGally (version 1.4.0), ggplot2 (version 3.0.0), ggnetwork (version 0.5.1), and intergraph (version 2.0.2).<br><br>All other analyses were performed in RStudio with the basic functions of the R programming language.<br><br>Rarefaction, extrapolation, and taxonomic richness analyses were performed with the open access EstimatesS software.

For manuscripts utilizing custom algorithms or software that are central to the research but not yet described in published literature, software must be made available to editors/reviewers. We strongly encourage code deposition in a community repository (e.g. GitHub). See the Nature Research guidelines for submitting code & software for further information.

Data

Policy information about availability of data

All manuscripts must include a data availability statement. This statement should provide the following information, where applicable:

- Accession codes, unique identifiers, or web links for publicly available datasets
- A list of figures that have associated raw data
- A description of any restrictions on data availability

The authors declare that the main data supporting the findings of this study are available within the article and its Supplementary Information files. The source data underlying all the figures and tables are provided as a Source Data file.

Field-specific reporting

Please select the one below that is the best fit for your research. If you are not sure, read the appropriate sections before making your selection.

☐ Life sciences ☐ Behavioural & social sciences ☒ Ecological, evolutionary & environmental sciences

For a reference copy of the document with all sections, see nature.com/documents/nr-reporting-summary-flat.pdf

Ecological, evolutionary & environmental sciences study design

All studies must disclose on these points even when the disclosure is negative.

Study description	We preformed a network analysis of fossil occurrence data. In the networks, fossil taxa are connected to co-occurring fossil taxa, their geologic formations, and/or their facies/paleoenvironments. We partitioned these networks into community units using established methods (e.g. label propagation, modularity optimization, random walks, etc.). We then used rarefaction, extrapolation, and taxonomic richness estimators to assess the diversity levels of the community units.
Research sample	Samples are fossil collection, as defined by the Paleobiology Database (i.e. a collection represents a set of fossil occurrences co-located at a unique point in geographic and stratigraphic space). In short, the samples were the smallest associations of co-occurring fossils and taxa, which we could reliably compile from primary and secondary literature. The samples include all collections of Ediacaran macrofossils. We made every effort to exhaust all means of growing the dataset prior to conducting network analysis.
Sampling strategy	The original dataset on occurrences of Ediacaran macroscopic body fossils (n=1829) was developed through revision of previously published datasets (Muscente et al., 2018; Boag et al., 2016; Muscente et al., 2017) and incorporation of additional information from primary and secondary literature sources following search protocols for collection-level sampling set forth by the PBDB (https://paleobiodb.org/data1.1/). We made every effort to exhaust all means of growing the Ediacaran dataset prior to conducting network analysis. Additional data on Cambrian body fossils were accessed from the Paleobiology Database. We corrected for uneven sampling by performing a number of subsampling/resampling analyses and testing the robustness of the results.
Data collection	A.D.M., N.B., T.H.B., L.A.B., and M.G.M. collected data by reviewing primary and secondary literature.
Timing and spatial scale	Fossils collections were sampled from rocks around the world throughout the Ediacaran system of the geologic record.
Data exclusions	We made no attempts to revise taxonomic work, alter designations of taxa in reports, or assign names to any material of uncertain affinities. So, samples comprised of fossils of uncertain taxonomic placement were omitted from the dataset. Additionally, based on conventional practices, we did not include disc-shaped taxa or fossils that may be pseudomorphs, taphomorphs, microbial induced sedimentary structures, or junior synonyms in the analyses, except where noted, as they are not reliable index fossils.
Reproducibility	As discussed in the methods, we conducted a wide number of subsampling and resampling analyses, which were performed repeatedly (hundreds or thousands of times). We did not observe any change in the results. So, all attempts to repeat the analyses were successful.
Randomization	We performed a number of analyses involving randomization, as discussed in the methods section. In most cases, allocation was random. The main exception pertains to randomized networks. In these cases, the degree distributions of the networks were maintained, so the analyses replicated the presence of well-connected and poorly connected taxa.
Blinding	N/A

Did the study involve field work? ☐ Yes ☒ No

Reporting for specific materials, systems and methods

We require information from authors about some types of materials, experimental systems and methods used in many studies. Here, indicate whether each material, system or method listed is relevant to your study. If you are not sure if a list item applies to your research, read the appropriate section before selecting a response.

Materials & experimental systems

n/a	Involved in the study
☒	☐ Antibodies
☒	☐ Eukaryotic cell lines
☒	☐ Palaeontology
☒	☐ Animals and other organisms
☒	☐ Human research participants
☒	☐ Clinical data

Methods

n/a	Involved in the study
☒	☐ ChIP-seq
☒	☐ Flow cytometry
☒	☐ MRI-based neuroimaging
